# Clinical efficacy and safety of different video-assisted thoracoscopic surgery approaches for bullous lung resection: a systematic review and meta-analysis

**DOI:** 10.3389/fsurg.2026.1838672

**Published:** 2026-05-29

**Authors:** Ting Gao, Xiaopeng He, Peiwen Zhao

**Affiliations:** Department of Respiratory and Critical Medicine, Xianyang Central Hospital, Xianyang, Shaanxi, China

**Keywords:** video-assisted thoracoscopic surgery, bullous lung resection, primary spontaneous pneumothorax, meta-analysis, clinical efficacy, surgical safety

## Abstract

**Objective:**

This meta-analysis evaluates the perioperative safety and comparative efficacy of single-port versus multi-port video-assisted thoracoscopic surgery (VATS) for bullous lung resection in patients with primary spontaneous pneumothorax.

**Methods:**

A systematic search of PubMed, Embase, Cochrane Library, and Web of Science was conducted through August 2025 to identify cohort studies comparing single-port (conventional or modified) versus multi-port (double-/triple-port) VATS. Data synthesis was performed using Stata 17.0 with random-effects models to account for inter-study heterogeneity.

**Results:**

Thirteen cohort studies (1,151 patients) were included. Single-port VATS was associated with significantly reduced hospital stay [mean difference (MD) = 0.246 days; 95% CI, 0.075–0.416] and a reduction in postoperative pain (MD = 1.100; 95% CI, 0.528–1.671) compared with multi-port VATS. The incidence of chest wall paresthesia was also lower (risk ratio = 0.580; 95% CI, 0.432–0.779). No significant differences were observed in operative time, chest tube duration, stapler use, complications, or recurrence. Subgroup analysis showed that the abbreviated hospital stay was primarily driven by conventional single-port techniques, whereas modified techniques showed no significant difference.

**Conclusion:**

Single-port VATS for bullous lung resection is associated with accelerated recovery, attenuated postoperative pain, and a lower incidence of neurological sequelae compared to multi-port VATS, without increasing operative time, complication, or recurrence rates. It represents a safe and feasible alternative, although further randomized trials are needed to establish superiority.

## Introduction

Primary spontaneous pneumothorax (PSP) is a prevalent thoracic condition characterized by a notable male predominance (1). Its primary pathophysiology involves the rupture of lung surface bullae/blebs, facilitating the entry of air into the pleural space. This results in acute symptoms such as pleuritic chest pain and dyspnea. Severe cases may progress to tension pneumothorax, a life-threatening emergency necessitating immediate intervention (2). Initial management typically relies on tube thoracostomy; however, recurrence rates following conservative therapy remain high, ranging from 34% to 35% (3). This risk increases significantly with each subsequent episode, establishing surgical intervention as the definitive strategy for recurrence prevention. While traditional open thoracotomy effectively mitigates through bullae resection and pleurodesis, it is associated with substantial morbidity, including extensive surgical trauma, severe postoperative pain, prolonged hospital stay, and high chest wall paresthesia rates (4).

Over the past decade, video-assisted thoracic surgery (VATS) has largely replaced open thoracotomy as the gold standard for PSP management, primarily due to its superior profile of minimal invasiveness (5). Early VATS (triple-/double-port) demonstrated reduced hospitalization times and attenuated postoperative pain relative to thoracotomy; however, the requirement for multiple incisions maintained a significant risk of intercostal nerve trauma. Triple-port VATS is associated with a chest wall paresthesia rate 52.9% (6), a complication that substantially compromises long-term postoperative quality of life. Consequently, the indications for single-port VATS have transitioned from basic diagnostic procedures, such as biopsy and simple wedge resections, to complex anatomical interventions, including lobectomy, segmentectomy, and bronchovascular angioplasty) (7). However, for patients undergoing bullectomy specifically for the management of PSP, the incremental clinical advantages of further reducing the number of ports remain a subject of ongoing debate. Key areas of clinical uncertainty persist regarding whether single-port VATS definitively accelerates recovery and whether modified single-port techniques provide substantive advantages over conventional single-port approaches. Therefore, this systematic review and meta-analysis sought to rigorously evaluate the comparative efficacy and safety of profiles of single-port versus multi-port VATS within this specialized cohort. Postoperative hospital stay was defined as the primary endpoint, with secondary endpoints including operative time, chest tube duration, pain scores, recurrence rates, and postoperative morbidity.

Existing studies vary in modified single-port protocols and pleurodesis techniques; many studies fail to report critical intraoperative data, such as stapler utilization. The majority of published evidence is derived from small-scale, single-center retrospective cohorts, which often lack the requisite statistical power to provide definitive safety and efficacy profiles. To address these limitations, the present study systematically aggregates and synthesizes clinical data comparing single-port versus multi-port VATS for PSP, providing a higher level of evidence via a rigorous meta-analytical framework. The primary objective is to integrate evidence across different modified single-port approaches, thereby facilitating individualized surgical decision-making and minimally invasive technology optimization in institutions.

## Materials and methods

### Study design

This systematic review and meta-analysis were conducted in accordance with the Preferred Reporting Items for Systematic Reviews and Meta-Analyses (PRISMA 2020) guidelines (8). We performed a systematic review and meta-analysis comparing clinical efficacy and safety of single-port versus double-port/triple-port VATS for the bullous lung resection, with the research question defined via the Population, Intervention, Comparison, and Outcomes (PICO) framework:

- Population: Patients (age ≥14 years) with CT-confirmed bullous lungs (diameter >1 cm) and PSP requiring surgical bullectomy.
- Intervention: Experimental group received single-port VATS (≤3 cm skin incision), including conventional and modified approaches [e.g., Single-Incision Laparoscopic Surgery (SILS) port assistance, suture traction, chest wall pulley assistance].
- Comparison: Control group received double-port/triple-port VATS (≥2 incisions: double-port = 1 camera + 1 working port; triple-port = 1 camera + 2 working ports).
- Outcomes: Clinical efficacy (operation time, hospital stay, chest tube indwelling time, postoperative pain score, stapler count, patient satisfaction); safety (postoperative recurrence rate, complication incidence, chest wall paresthesia incidence).

### Study types

Retrospective cohort studies, prospective cohort studies, and randomized controlled trials were included. Conversely, exclusion criteria were predefined to omit case series, reviews, meta-analyses, animal experiments, *in vitro* studies, and comparative studies involving non-VATS approaches (e.g., open thoracotomy).

## Study population

### Diagnostic criteria

Bullous lungs (air-containing cysts from alveolar wall rupture/fusion, diameter >1 cm) confirmed by chest CT (slice thickness ≤1 mm) or radiography, with/without spontaneous pneumothorax (9).

### Inclusion criteria

- Age ≥14 years;
- No severe surgical contraindications;
- Confirmed diagnosis of PSP requiring surgical intervention; and
- Preoperative chest CT evidence of bullae/blebs as the etiology of the pneumothorax.

### Exclusion criteria

- Comorbidity with other lung diseases (e.g., lung cancer, pulmonary tuberculosis, pulmonary fibrosis);
- Pregnancy or lactation;
- A history of previous thoracic surgery; and
- Concurrent performance of other thoracic surgeries (e.g., lobectomy, segmentectomy).

### Intervention and control measures

Both groups used consistent core procedures: bullous resection (wedge resection with linear stapler/suture ligation) and pleurodesis.

- Experimental Group (Single-port VATS): All operations via a single ≤3 cm incision, classified as: (1) Conventional single-port-no auxiliary devices; (2) Modified single-port-use of any auxiliary device (e.g., SILS port, wound protector, suture traction, chest wall pulley).
- Control Group (Double-port/Triple-port VATS): Thoracoscope/instruments via ≥2 incisions: double-port (1 camera + 1 working port); triple-port (1 camera + 2 working ports).

## Outcomes

### Clinical efficacy indicators

(a) Operation time;
(b) Hospital stay;
(c) Chest tube indwelling time;
(d) Postoperative pain score;
(e) Patient satisfaction; and
(f) Number of staplers used.

### Safety indicators

(a) Postoperative recurrence rate;
(b) Incidence of chest wall paresthesia; and
(c) Incidence of postoperative complications.

### Literature search strategy

A comprehensive, systematic literature search was conducted from database inception to August 2025 across PubMed, Embase, Cochrane Library, and Web of Science. Search terms covered the following three categories: bullous resection (e.g., bullectomy, lung bulla resection); thoracoscopic surgery (e.g., single-port VATS, double-/triple-port VATS); and pneumothorax (e.g., spontaneous pneumothorax, primary spontaneous). Detailed strategies are in Supplementary Table S1.

### Literature screening and data extraction

Two independent reviewers (Ting Gao and Xiaopeng He) conducted initial screening to exclude ineligible studies, followed by secondary full-text screening. Discrepancies regarding study eligibility were resolved by the third reviewer, Peiwen Zhao. The selection process was documented in a PRISMA 2020 flow diagram (total retrieved, duplicates removed, excluded at each stage, and finally included).

Data extraction parameters encompassed basic info (first author, year, study type, sample sizes, age, interventions, follow-up) and outcome data (raw values for efficacy and safety indicators).

### Extracted data

Basic study information were extracted for each included record, including first author, publication year, study type, sample sizes of the experimental and control groups, age of patients, intervention measures (specific types of single-port/modified single-port VATS, specific incision distribution, and operational details of double-port/triple-port VATS), and the duration of clinical follow-up.

Outcome indicator data: Raw data of clinical efficacy indicators encompassed: operation time, hospital stay, chest tube indwelling time, postoperative pain score, number of staplers used, and patient satisfaction; and safety indicators specifically postoperative recurrence rate, incidence of postoperative complications, and incidence of chest wall paresthesia.

### Risk of bias assessment

The Newcastle–Ottawa Scale (NOS) (10) assessed bias across three dimensions (eight items) with scores 0–9: ≥ 6 = low bias (included in meta-analysis); <6 = high bias (only in sensitivity analysis). Dimensions:

- Population selection (clarity of inclusion/exclusion criteria, control group rationality, exposure measurement accuracy);
- Group comparability (confounder adjustment, e.g., age, gender, bulla type);
- Outcome measurement (assessment objectivity, follow-up adequacy/completeness).

### Statistical analysis methods

Analyses used Stata 17.0 (two-tailed *α* = 0.05). Effect sizes:

- Continuous outcomes with consistent units (hospital stay, operation time, chest tube indwelling time, postoperative pain score, stapler quantity): Mean Difference (MD).
- Continuous outcomes with inconsistent measurement tools (patient satisfaction): Standardized Mean Difference (SMD).
- Dichotomous outcomes: Risk ratio (RR).

All effect sizes included 95% confidence intervals (95% CI). MD/SMD <0 or RR < 1 indicated experimental group superiority.

Heterogeneity was evaluated via Cochran’s *Q*-test (*α* = 0.10) and I^2^:

- I^2^ < 25%: Low heterogeneity (fixed-effects model);
- 25% ≤ I^2^ ≤ 50%: Moderate heterogeneity (fixed-effects model if minimal clinical heterogeneity);
- I^2^ > 50%: High heterogeneity (random-effects model + subgroup analysis: conventional vs. modified single-port VATS).

Sensitivity analysis used the “leave-one-out” method (excluded one study at a time, re-pooled effect sizes) and the exclusion of high-bias studies (NOS <6); stability was confirmed if the effect size change was <10% and 95% CI excluded 1 (RR) or 0 (MD/SMD).

Publication bias (for ≥10 studies) was assessed via funnel plots (visual symmetry) and Egger’s/Begg’s tests; *P* > 0.05 indicated no significant bias.

## Results

### Literature search and screening results

**Figure 1:**
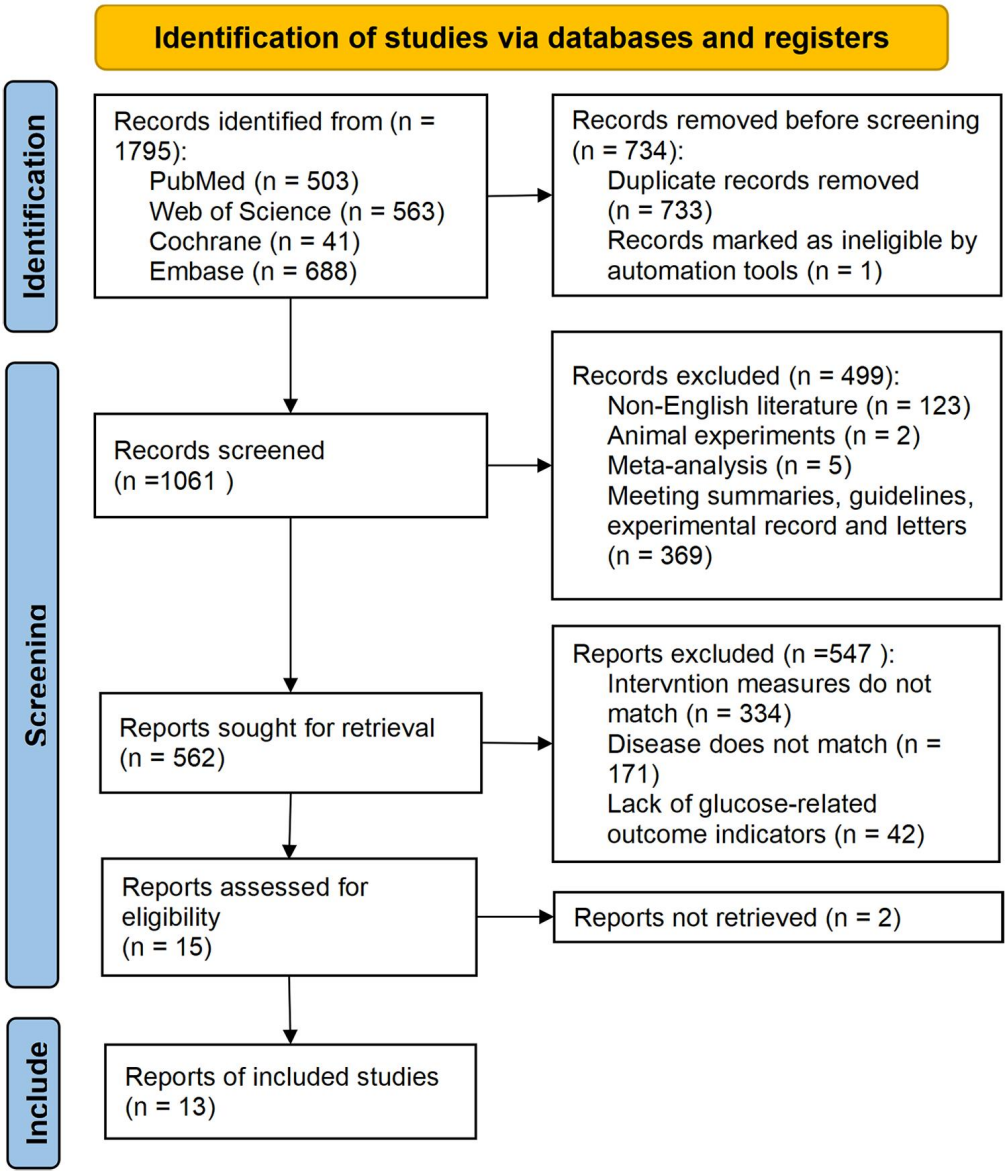
Flow diagram of literature screening process according to PRISMA 2020.

A total of 1,795 studies were initially identified through database searching, of which 1 was automatically excluded by the system for failing basic database criteria. After duplicate removal via EndNote X9, 1,061 studies remained. Initial screening (by title/abstract) excluded 123 non-English or non-bullous resection studies, 2 animal experiments, 5 meta-analyses, and 369 documents (conference abstracts, guidelines, etc.), leaving 562 studies. Full-text secondary screening excluded 42 studies with incomplete data, 334 with inconsistent interventions, 171 with ineligible patients, and 2 studies that were unrecoverable. Finally, 13 studies were included (11–23). The comprehensive study selection workflow is illustrated in the PRISMA 2020 flow diagram in Figure 1.

### Basic characteristics of included studies

Thirteen cohort studies involving 1,151 patients (617 experimental, 534 control) were included in the final analysis. Geographically, most were from Asia: three from Japan (12, 13, 20), two from China (16, 21), three from South Korea (11, 18, 19); and others were from Italy [two (15, 22)], the UK [one (14)], the Netherlands [one (23)], and Turkey [one (17)]. Comprehensive baseline characteristics and clinical demographic data are summarized in Tables 1, 2.

**Table 1:** Basic information of included studies.

Study	Author (Year)	Country	Study type	Age of control group	Age of the experimental group	Sample size of the control group	Sample size of the intervention group	Control group intervention measures:	Intervention measures for the experimental group	Follow-up period
1	Yang et al.（2013）	South Korea	Retrospective cohort study	27.1 ± 12.3 years	22.0 ± 9.1 years	13	27	Traditional three-port VATS: 3 incisions (11 mm incision on the midaxillary line at the 7th/8th ribs for placing the thoracoscope, 2 cm incision on the anterior axillary line at the 4th/5th ribs + 5 mm incision on the posterior axillary line at the 6th/7th ribs for placing the surgical instruments), removal of pulmonary bullae + pleural friction fixation, using 2 disposable trocars	Single-port VATS (SITS): 2.5 cm incision (at the 5th intercostal space along the midaxillary line), insert SILS multi-channel flexible port, placed in a “inverted triangle” layout with 5 mm 30° thoracoscopes, rotatable forceps and suturing instrument, reinforce the pulmonary bulla after resection with fibrin glue, and fix the pleura by friction	Control group: 4.6 (2.3–8.2) months; Experimental group: 3.5 (2.1–7.0) months
2	Hazama et al.（2003）	Japan	Retrospective cohort study	27.6 ± 11.8 years (15–56 years)	24.4 ± 12.0 years (14–70 years)	54	60	Traditional VATS: 3 incisions (10 mm incision on the midaxillary line at the 6th rib for placing the thoracoscope, 5 mm incision on the anterior axillary line at the 5th rib + 10 mm incision on the anterior axillary line at the 7th rib for placing the surgical instruments), using a 10 mm linear suture device to remove pulmonary bullae (diameter >2 cm), Nd:YAG laser ablation of the surrounding lesions of the bullae	Needle-type VATS: 3 2 mm incisions (at the 3rd/5th/6th intercostal spaces), insert 2 mm thoracoscopes, ultrafine forceps and Nd:YAG laser fiber, only laser ablation of pulmonary bulla (diameter ≤2 cm), no resection of lung tissue, postoperative use 2 mm ultrafine thoracic tube for drainage	4–45 months
3	Tsuboshima et al.（2015）	Japan	Retrospective cohort study	25.9 ± 8.8 years	22.9 ± 8.7 years	35	34	Traditional three-port VATS (cVATS): 3 incisions (5.5/11.5 mm incisions on the midaxillary line at the 6/7th ribs for placing the thoracoscope, 5.5 mm incision on the anterior axillary line at the 4th rib + 5.5/11.5 mm incisions on the anterior axillary line at the 6/7th ribs for placing the surgical instruments), removal of pulmonary bullae + fixation with Surgicel Nu-Knit using a surgical instrument	Single-port VATS (PulLE): 1 17 mm–25 mm incision (at the 6th intercostal space along the midaxillary line), use 2–0 nylon thread to suture the visceral pleura (to pull the lesion) and parietal pleura (to form a chest wall pulley), adjust the position of the pulmonary bulla after traction and then resect, use Surgicel Nu-Knit to reinforce the suture line	Control group: 19.9 months; Experimental group: 6.7 months
4	Jutley et al.（2005）	United Kingdom	Retrospective cohort study	32.1 ± 10 years	28.9 ± 15.4 years	19	16	Traditional three-port VATS: 3 incisions (1–1.5 cm incisions arranged in a “reverse triangle”, 5/10 mm incision on the midaxillary line at the 7th rib for placing the thoracoscope, 5/10 mm incisions on the inferior margin of the scapula—anterior axillary line at the 5th rib), removal of pulmonary bullae using an Endo-GIA suture device, pleural friction to pleural debridement	Single-port VATS: 2–2.5 cm incision (at the 5th intercostal space along the posterior axillary line), insert 5 mm 0° thoracoscopes and 2 rotatable instruments (Endo-GIA Universal, Roticulator Endograsp), resection of pulmonary bulla + pleural friction to pleural stripping	Control group: 32.1 ± 9.9 months; Experimental group: 9.4 ± 6.6 months
5	Salati et al.（2008）	Italy	Retrospective cohort study	26.4 ± 6.4 years	24 ± 6.3 years	23	28	Traditional three-port VATS: 3 incisions (10 mm incision on the midaxillary line at the 7th rib for placing the thoracoscope, 2 incisions on the anterior axillary line at the 5th rib for placing the surgical instruments), removal of pulmonary bullae using a surgical instrument, pleural friction fixation, chest tube placed through the thoracoscopic port	Single-port VATS: 2.5 cm incision (at the 5th intercostal space along the midaxillary line), insert 5 mm 30° thoracoscopes and 2 5 mm instruments/suturing devices, resection of pulmonary bulla + pleural friction fixation, thoracic tube placed through the surgical incision	Control group: 39 months; Experimental group: 13 months
6	Chen et al.（2012）	Taiwan, China	Retrospective cohort study	24.5 years	29.1 years	26	36	Traditional three-port VATS: 3 incisions (12–15 mm incisions at the 6–7th ribs for placing the thoracoscope, 5 mm incision on the anterior axillary line at the 5th rib for placing the grasping instrument, 12 mm–15 mm incision at 1 inch below the inferior margin of the scapula on the anterior axillary line for placing the linear suture device), wedge-shaped removal of pulmonary bullae + mechanical pleural fixation, chest tube (24–28Fr) for drainage	Single-port VATS: 15 mm incision (between the 5th/6th intercostal spaces along the midaxillary line—axillary anterior line), insert 5 mm 30° thoracoscopes, forceps and linear suturing instrument, no trocar, wedge resection of pulmonary bulla + mechanical pleural fixation, thoracic tube placed through the incision	Control group: 30.5 months; Experimental group: 16.3 months
7	Ocakcioglu et al.（2015）	Turkey	Prospective cohort study	Two-port group: 27.4 years; Three-port group: 27.9 years	Single-chamber group 26.2 years	40	37	Three-port VATS: 3 incisions (10 mm incision on the midaxillary line at the 7/8th ribs for placing the thoracoscope, 5 mm incision on the anterior axillary line at the 4th/5th ribs + 5 mm incision on the posterior axillary line at the 6th/7th ribs for placing the surgical instruments), the operation is the same as the two-port group	Single-port VATS: 20–25 mm incision (at the 5th/6th intercostal spaces along the midaxillary line), insert 30°/10 mm thoracoscopes and operating instruments, resection of pulmonary bulla + pleural friction fixation, thoracic tube placed through the incision	The specific mean value was not clear, only the period of follow-up was mentioned
8	Kang et al.（2014）	South Korea	Retrospective cohort study	3.79 ± 1.44 days (reference for chest tube drainage time)	2.82 ± 0.92 days (reference for chest tube drainage time)	19	33	Traditional three-port VATS: 3 incisions (5 mm incision on the midaxillary line at the 7th rib for placing the thoracoscope, 12 mm incision on the anterior axillary line at the 6th rib + 5 mm incision on the posterior axillary line at the 5th rib for placing the surgical instruments), removal of pulmonary bullae/pulmonary apex wedge-shaped removal + pleural friction fixation, chest tube (16–20Fr) for drainage	Single-port VATS: 2.10 ± 0.28 cm incision (at the 5th intercostal space along the anterior axillary line), use X-shaped wound retractor to protect nerves, insert 5 mm 30° thoracoscopes and operating instruments, operate as in the three-port group, thoracic tube placed through the incision	Three-port group: 15.0 months; Single-port group: 14.0 months
9	Song et al.（2015）	South Korea	Retrospective cohort study	23.4 ± 6.4 years	22.0 ± 6.3 years	23	37	Traditional three-port VATS: 3 incisions (15 mm incision on the midaxillary line at the 7th rib for placing the thoracoscope, 5 mm incision on the anterior axillary line at the 5th rib + 5 mm incision on the posterior axillary line at the 6th/7th ribs for placing the surgical instruments), removal of pulmonary bullae + pleural friction fixation, chest tube (15 mm incision) placed	Single-port VATS (SITS): 2.5 cm incision (at the 4th−6th intercostal spaces along the midaxillary line), insert ENDO KEEPERTM wound protector, 5 mm 30° thoracoscopes and operating instruments, resection of pulmonary bulla + fibrin glue spraying, thoracic tube placed through the incision	Control group: 3.1 (2.1–10.2) months; Experimental group: 2.9 (1.8–12.5) months
10	Yoshikawa et al.（2021）	Japan	Retrospective cohort study	22 ± 6.4 years	23 ± 6.2 years	71	161	Traditional three-port VATS (3P-VATS): 3 incisions (15 mm incision on the anterior axillary line at the 5th rib for placing the suture device, 7 mm incision on the posterior axillary line at the 5/6th ribs for placing 5 mm flexible thoracoscope, 5 mm incision on the anterior axillary line at the 3rd rib for placing the grasping instrument), removal of pulmonary bullae + PGA patch + fibrin glue fixation	Single-port VATS (U-VATS): 25 mm–30 mm incision (at the 5th/6th intercostal spaces along the anterior axillary line), insert 1–3 cm wound protector and 5 mm flexible thoracoscopes, operate as in the three-port group, when necessary add 3 mm auxiliary incision	The specific mean value of the groups was not clear, the overall follow-up was ≥ 2 years
11	Wang et al.（2020）	China	Non-randomized retrospective study	25.52 ± 0.64 years	23.91 ± 0.68 years	48	56	Double-port VATS: 2 incisions (10 mm incision on the midaxillary line at the 7th/8th rib intercostal space for placing the thoracoscope, 2.0 cm incision on the anterior axillary line at the 3rd rib intercostal space for placing the surgical instruments), resection of pulmonary bullae/wedge resection of the pulmonary apex + pleural friction fixation, chest tube placed through the incision at the 7th/8th rib intercostal space	Single-port VATS: 2.5 cm incision (at the 5th intercostal space along the anterior axillary line), insert disposable wound protector and 10 mm 30° thoracoscopes, operating instruments, operate as in the double-port group, thoracic tube placed through the 4th intercostal incision	23.23 ± 0.83 months; Double-port group: 25.06 ± 1.00 months
12	Fiorelli et al.（2020）	Italy	Retrospective cohort study	35 ± 1.8 years	34 ± 2.6 years	22	21	Traditional three-port VATS: 3 incisions (10 mm incision on the midaxillary line at the 7th rib intercostal space for placing the thoracoscope, 15 mm incision on the anterior axillary line at the 5th rib intercostal space + 5 mm incision on the subscapular line at the 5th rib intercostal space for placing the surgical instruments), resection of pulmonary bullae + pleural friction fixation, chest tube placed through the port at the thoracoscopic end	Modified single-port VATS (suture traction): 2 cm–2.5 cm incision (at the 5th intercostal space along the anterior axillary line), use 0-number suture to suture the visceral/parietal pleura to form traction, insert 30° thoracoscopes and operating instruments, resection of pulmonary bulla + pleural friction fixation, thoracic tube placed through the incision	The specific mean value was not clear, there was no recurrence during the follow-up period
13	Janssen et al.（2024）	the Netherlands	Retrospective cohort study	39.0 (26–54) years (median, IQR)	36.4 (22–54) years (median, IQR)	141	71	Multi-port VATS (mVATS): 3 incisions (12 mm incision on the midaxillary line at the 9th rib intercostal space for placing the thoracoscope, 12 mm incision on the anterior axillary line at the 7/8th rib intercostal space + 5 mm trocar incision on the subscapular line at the 6th rib intercostal space for placing the surgical instruments), resection of pulmonary bullae/wedge resection of the pulmonary apex + parietal pleural stripping	Single-port VATS (uVATS): A 3–4 cm incision (between the midaxillary line and the anterior axillary line at the 4th/5th intercostal space), a thoracoscope and surgical instruments are inserted, and the operation is the same as the multi-port group. The chest tube is placed through the incision.	Control group: 825 (203–3,126) days; Experimental group: 247 (97–1,456) days

**Table 2:** Outcome indicators of included studies.

Study	Author (year)	Clinical outcome indicators	Safety indicators	Secondary supplementary indicators
1	Yang et al.（2013）	Surgery duration, hospital stay, postoperative pain (VAS score), recurrence rate	Abnormal sensation rate, complications (chemical pleurodesis, reoperation, wound infection)	Surgical material cost, satisfaction with wound scar
2	Hazama et al.（2003）	Surgery duration, chest tube retention time, hospital stay, recurrence rate	Complications (prolonged air leakage, refractory intercostal pain)	Postoperative use rate of painkillers (non-steroidal anti-inflammatory drugs), serum creatine phosphokinase level
3	Tsuboshima et al.（2015）	Surgery duration, postoperative chest tube retention time, hospital stay, recurrence rate	Blood loss, complications	Number of suturing devices used, number of pulmonary bullae, number of removed lung tissue, surgical scar condition
4	Jutley et al.（2005）	Chest tube retention time, hospital stay, hospital stay pain score (VAS 0–4 points), residual pain score, recurrence rate	Sensation abnormality rate (numbness, tingling, changes in breast sensitivity, etc.)	Volume of removed tissue, age, lung function (FEV₁.₀%, FVC%)
5	Salati et al.（2008）	Surgery duration, postoperative hospital stay, chronic pain (numerical pain score, McGill pain questionnaire), recurrence rate	Sensation abnormality rate, rate of prolonged air-leak (>5 days)	Surgical cost (surgical instrument cost, operating room occupancy cost, postoperative hospitalization cost), normal activity recovery rate
6	Chen et al.（2012）	Surgery duration, hospital stay, postoperative pain (VAS score), recurrence rate	Complications (wound infection)	ICU stay time, surgical side, surgical indication (recurrence, prolonged air leak, hemothorax)
7	Ocakcioglu et al.（2015）	Surgery duration, chest tube retention time, hospital stay, postoperative pain (VAS score), recurrence rate	Complications (conversion to thoracotomy, 30-day mortality rate)	Total postoperative drainage volume, patient satisfaction score
8	Kang et al.（2014）	Surgery duration, chest tube retention time, hospital stay, postoperative pain (VAS score), recurrence rate	Postoperative sensation abnormality rate, complications	Number of wedge resections, 24-h postoperative thoracic tube drainage volume, size of surgical scar
9	Song et al.（2015）	Surgery duration, chest tube retention time, hospital stay, postoperative pain (VAS score), recurrence rate	Complications (air leakage, pleural effusion)	Number of wedge resections, incidence of pleural adhesion, number of additional intramuscular analgesic drug uses
10	Yoshikawa et al.（2021）	Surgery duration, chest tube retention time, hospital stay, recurrence rate	Intraoperative blood loss, complications (wound infection)	Rate of conversion to three-port VATS, number of pulmonary bullae, age, impact of number of pulmonary bullae on recurrence (multivariate analysis)
11	Wang et al.（2020）	Surgery duration, chest tube retention time, hospital stay, postoperative pain (VAS score), recurrence rate	Intraoperative blood loss, sensation abnormality rate, complications (air leakage, pleural effusion, wound infection)	Number of suturing devices used, patient satisfaction score
12	Fiorelli et al.（2020）	Surgery duration, chest tube retention time, hospital stay, postoperative pain (VAS score), recurrence rate	Intraoperative blood loss, sensation abnormality rate, complications	Number of postoperative drainage volumes, patient satisfaction score
13	Janssen et al.（2024）	Surgery duration, chest tube retention time, hospital stay, recurrence rate	Complications (classified according to Clavien-Dindo scale)	Surgical type (pulmonary bullae resection/wedge resection), rate of conversion to thoracotomy/conversion to multi-port, ICU admission rate, 30-day readmission rate, age, ASA classification on prognosis (multivariate analysis)

### Risk of bias assessment results of included studies

All 13 included studies were cohort studies, with Newcastle–Ottawa Scale (NOS) total scores ranging from 7 to 9. All studies were classified as high-quality cohort studies; no moderate- or low-quality studies were identified, indicating an overall low risk of bias (see Table 3).

**Table 3:** NOS scale scores of the 13 included studies.

Study No.	Selection (scores of items 1–4)	Comparability (scores of items 5–6)	Outcome (scores of items 7–9)	Total score	Quality grade
1	3.5	1.5	3	8	High quality
2	3.5	2	3	8.5	High quality
3	3.5	1.5	2.5	7.5	High quality
4	3.5	1	2.5	7	High quality
5	3.5	2	3	8.5	High quality
6	3.5	2	3	8.5	High quality
7	4	2	3	9	High quality
8	3.5	2	3	8.5	High quality
9	3.5	1.5	2.5	7.5	High quality
10	3.5	2	3	8.5	High quality
11	3.5	2	3	8.5	High quality
12	3.5	1.5	2.5	7.5	High quality
13	4	2	3	9	High quality

### Detailed assessment results

Selection bias: The exposure factors were objectively recorded in all studies, and the control group was selected using the same method as the experimental group. All outcomes occurred postoperatively, resulting in an extremely low risk of selection bias.

Outcome measurement bias and attrition bias:

- utcome assessment was derived from hospital medical record systems or independent evaluations in 11 studies (11, 12, 14–21, 23);
- The follow-up duration was ≥6 months in 10 studies (12, 14–18, 20–23);
- The follow-up completion rate was ≥90% in eight studies (12, 15–18, 20, 21, 23).
- These results indicated low risks of outcome measurement bias and attrition bias.

Confounding bias: Only three studies (14, 19, 22) reported baseline balance without statistical adjustment, which may have led to uncontrolled confounding.

## Meta-Analysis results of clinical efficacy and safety comparison

### Comparison of operation duration

Subgroup analyses were performed according to the clearly defined classification of conventional versus modified single-port VATS. This meta-analysis compared operation duration between single-port and triple-port VATS, with subgroup and overall results as follows:

- Conventional single-port subgroup: 7 studies included (11, 12, 15–18, 20, 21, 23). Pooled MD = 0.267 (95% CI: −0.270 to 0.805, z = 0.976, *P* = 0.329).
- Modified single-port subgroup: three studies included (13, 19, 22). Pooled MD = −0.224 (95% CI: −0.665 to 0.217, z = −0.996, *P* = 0.319).
- Overall analysis: Pooled MD = 0.134 (95% CI: −0.290 to 0.558, z = 0.619, *P* = 0.536).

All *P* > 0.05, indicating no significant difference in operation duration (Supplementary Figure S1).

### Heterogeneity analysis

- Conventional single-port subgroup: High heterogeneity (Cochran's Q = 93.407, *P* < 0.05, I^2^ = 92.5%).
- Modified single-port subgroup: Moderate heterogeneity (Cochran's Q = 4.102, *P* = 0.129, I^2^ = 51.2%).
- Overall analysis: High heterogeneity (Cochran's Q = 102.091, df = 9, *P* < 0.05, I^2^ = 90.2%).
- Subgroup heterogeneity test (Q = 1.92, *P* = 0.166) showed stratification did not explain overall heterogeneity.

### Sensitivity and publication bias analysis

Sensitivity analysis (sequential exclusion of each study) showed minimal fluctuations in the pooled effect size, confirming the robustness of the result (Supplementary Figure S2).

No publication bias was found via Egger’s test (*P* = 0.624) or Begg’s test (*P* = 0.938); the funnel plot is in Supplementary Figure S3.

### Comparison of hospital stay duration

This meta-analysis compared hospital stay between single-port and triple-port VATS, with subgroup and overall results:

- Conventional single-port group: 10 studies (11, 12, 14–18, 20, 21, 23). Pooled MD = 0.298 (95% CI: 0.086–0.510, z = 2.756, *P* = 0.006 < 0.05), indicating significantly shorter stay vs. triple-port.
- Modified single-port group: 3 studies (13, 19, 22). Pooled MD = 0.071 (95% CI: −0.231–0.373, z = 0.462, *P* = 0.644 > 0.05), no significant difference.
- Overall analysis: Pooled MD = 0.246 (95% CI: 0.075–0.416, z = 2.823, *P* = 0.005), overall shorter stay in single-port (Supplementary Figure S4).

### Heterogeneity analysis

- Conventional single-port: Moderate-to-high (Q = 17.17, *P* = 0.028, I^2^ = 53.4%).
- Modified single-port: No heterogeneity (Q = 0.41, *P* = 0.815, I^2^ = 0.0%).
- Overall: Moderate (Q = 18.80, *P* = 0.055, I^2^ = 41.5%).

The subgroup test (Q = 1.45, *P* = 0.228) showed stratification that did not explain overall heterogeneity.

### Sensitivity and publication bias analysis

To address the concern that the modified single-port group contained heterogeneous techniques (SILS port, wound protector, traction sutures), we performed two additional sensitivity analyses. First, after excluding all modified single-port studies (i.e., analyzing only conventional single-port versus multi-port), the difference remained significant (MD = –0.214, 95% CI −0.391 to −0.038, *P* = 0.017; Supplementary Figure S4a). Second, after excluding the wound-protector study (19), the pooled estimate was still significant (MD = –0.184; 95% CI, −0.316 to −0.052, *P* = 0.006; Supplementary Figure S4b). These results indicate that the overall finding of a shorter hospital stay with single-port VATS is robust and not driven by any specific modified technique.

Sensitivity analysis confirmed result robustness (minimal effect size fluctuations, significant conclusion unchanged; Supplementary Figure S5). No bias via Egger's (*P* = 0.575) or Begg's (*P* = 0.945) tests (funnel plot: Supplementary Figure S6).

### Postoperative recurrence rate

Comparison of recurrence rate between single-port and triple-port VATS:

- Conventional single-port group: 10 studies (11, 12, 14–18, 20, 21, 23). Pooled RR = 0.636 (95% CI: 0.307–1.318, z = −1.217, *P* = 0.224).
- Modified single-port group: 3 studies (13, 19, 22). Pooled RR = 0.949 (95% CI: 0.423–2.132, z = −0.126, *P* = 0.900).
- Overall analysis: Pooled RR = 0.761 (95% CI: 0.443–1.308, z = −0.989, *P* = 0.323), no significant difference (Supplementary Figure S7).

### Heterogeneity analysis

No heterogeneity in conventional (Q = 2.35, *P* = 0.938, I^2^ = 0.0%), modified (Q = 0.62, df = 2, *P* = 0.735, I^2^ = 0.0%), or overall (Q = 3.48, *P* = 0.968, I^2^ = 0.0%) analyses.

Subgroup tests: No significant differences in effects for complications (Q = 0.50, *P* = 0.480) or recurrence (Q = 0.52, df = 1, *P* = 0.471).

### Sensitivity and publication bias analysis

Sensitivity analysis confirmed robustness (Supplementary Figure S8). No bias via Egger's (*P* = 0.485) or Begg's (*P* = 0.533) tests (funnel plot: Supplementary Figure S9).

### Postoperative complications

Comparison of complication incidence:

- Conventional single-port group: 6 studies (11, 12, 17, 20, 21, 23). Pooled RR = 0.588 (95% CI: 0.338–1.024, z = −1.876, *P* = 0.061), no significant difference.
- Modified single-port group: 2 studies (19, 22). Pooled RR = 0.819 (95% CI: 0.393–1.705, z = −0.534, *P* = 0.594), no significant difference.
- Overall analysis: Pooled RR = 0.673 (95% CI: 0.449–1.009, *P* = 0.055), indicating no statistically significant difference between single-port and multi-port VATS (Supplementary Figure S10).
- Heterogeneity: No heterogeneity in conventional (Q = 4.46, df = 5, *P* = 0.486, I^2^ = 0.0%) or overall (Q = 6.04, *P* = 0.535, I^2^ = 0.0%) analyses; low in modified (Q = 1.09, *P* = 0.296, I^2^ = 8.5%).

Sensitivity/Publication Bias: Sensitivity analysis confirmed robustness (core conclusion unchanged; Supplementary Figure S11). No bias via Egger's (*P* = 0.528) or Begg's (*P* = 0.711) tests (funnel plot: Supplementary Figure S12).

### Incidence of chest wall paresthesia

Six studies (11, 14, 15, 18, 21, 22) compared chest wall paresthesia. All RRs <1 (consistently lower risk in single-port). Overall pooled RR = 0.580 (95% CI: 0.432–0.779, z = −3.615, *P* < 0.05), indicating 42% lower risk in single-port versus triple-port (Supplementary Figure S13).

Heterogeneity: Q = 2.62 (*P* = 0.759), I^2^ = 0.0% (high consistency).

Sensitivity/Publication Bias: Sensitivity analysis confirmed stability (conclusion unchanged; Supplementary Figure S14). Egger's test (*P* = 0.049 < 0.05) and Begg's test (*P* = 0.133) suggested minimal impact on robustness.

### Comparison of postoperative pain score (VAS)

Nine studies (11, 14–19, 21, 22) compared VAS scores. Overall pooled MD = 1.100 (95% CI: 0.528–1.671, z = 3.772, *P* < 0.001), indicating significantly lower pain in single-port (Supplementary Figure S15).

Heterogeneity: High in conventional (Q = 66.86, *P* < 0.001, I^2^ = 91.0%) and overall (Q = 69.06, *P* < 0.05, I^2^ = 88.4%) analyses; no heterogeneity in modified (Q = 0.24, *P* = 0.625, I^2^ = 0.0%).

The subgroup test (Q = 0.55, *P* = 0.459) showed that stratification did not explain overall heterogeneity.

Sensitivity/Publication Bias: Sensitivity analysis confirmed robustness (significant conclusion unchanged; Supplementary Figure S16). No bias via Egger's (*P* = 0.841) or Begg's (*P* = 0.348) tests (funnel plot: Supplementary Figure S17).

## Secondary outcome indicators

### Comparison of intraoperative stapler quantity

Three studies (13, 21, 22) compared the use of intraoperative staplers. Overall pooled MD = 0.132 (95% CI: −0.977–1.241, z = 0.233, *P* = 0.815 > 0.05), indicating no significant difference (Supplementary Figure S18).

### Heterogeneity analysis

Heterogeneity analysis results showed Cochran’s Q = 30.32 (*P* < 0.05) and I^2^ = 93.4%, indicating extremely high heterogeneity among the included studies.

### Publication bias analysis

No statistical evidence of publication bias was found via Egger’s test (*P* = 0.502) and Begg’s test (*P* = 0.602).

### Chest tube indwelling time

Comparison of chest tube indwelling time between single-port and triple-port VATS:

- Conventional single-port group: 7 studies (12, 14, 17, 18, 20, 21, 23). Pooled MD = 0.312 (95% CI: −0.046–0.670, z = 1.707, *P* = 0.078), no significant difference.
- Modified single-port group: 3 studies (13, 19, 22). Pooled MD = 0.049 (95% CI: −0.253–0.352, z = 0.319, *P* = 0.749 > 0.05), no significant difference.
- Overall analysis: Pooled MD = 0.242 (95% CI: −0.030–0.514, z = 1.747, *P* = 0.081), no significant difference (Supplementary Figure S19).

### Heterogeneity analysis

- Conventional single-port: High (Q = 33.67, *P* < 0.05, I^2^ = 82.2%).
- Modified single-port: No heterogeneity (Q = 1.05, *P* = 0.593, I^2^ = 0.0%).
- Overall: High (Q = 35.84, *P* < 0.05, I^2^ = 74.9%).

The subgroup test (Q = 1.21, *P* = 0.272) that showed stratification did not explain overall heterogeneity.

Publication Bias: No evidence via Egger’s (*P* = 0.475) or Begg’s (*P* = 0.858) tests.

### Postoperative patient satisfaction

Three studies (17, 21, 22) compared postoperative satisfaction. Overall pooled SMD = 1.480 (95% CI:−0.096 to 3.057, *P* = 0.066), with extremely high heterogeneity (I^2^ = 96.4%). Only three studies reported patient satisfaction, and no statistically significant difference was detected between the two approaches (Supplementary Figure S20).

### Heterogeneity analysis

Heterogeneity analysis results showed Cochran's Q = 55.47 (*P* < 0.05) and I^2^ = 96.4%, indicating extremely significant and extremely high heterogeneity among the included studies. It was hypothesized that this heterogeneity might be attributable to differences in the measurement tools used for patient satisfaction.

### Publication bias analysis

No statistical evidence of publication bias was found via Egger’s test (*P* = 0.588) and Begg’s test (*P* = 0.602).

## Discussion

This meta-analysis of 13 cohort studies (1,151 patients) evaluated the comparative perioperative performance of single-port versus multi-port VATS for bullectomy in the setting of primary spontaneous pneumothorax. The pooled results indicate that single-port VATS is associated with a significantly shorter hospital stay, lower postoperative pain scores, and reduced incidence of chest wall paresthesia. No statistically significant differences were observed for operative duration, duration of chest tube drainage, stapler use, patient satisfaction, postoperative complications, or recurrence rates. These findings suggest selective advantages of single-port VATS in recovery and nerve preservation, while simultaneously confirming therapeutic non-inferiority in other clinical and safety outcomes.

### Advantages in postoperative recovery and nerve protection

Triple-port VATS requires incisions across multiple intercostal spaces, which may damage intercostal neurovascular bundles. In the present meta-analysis, the single-port group had a significantly lower incidence of chest wall paresthesia compared to the multi-port group (pooled RR = 0.58, 95% CI: 0.43–0.78, *P* < 0.001), with no subgroup differences between conventional and modified single-port techniques. This finding supports the hypothesis that reducing the number of incisions fundamentally is associated with a lower risk of intercostal nerve traction or thermal injury (5).

Regarding postoperative recovery, single-port VATS was associated with a significantly shorter length of stay (MD = 0.246, *P* = 0.005), which may be partially attributed to reduced postoperative pain (pooled MD = 1.100, *P* < 0.001). However, chest tube indwelling time did not differ significantly between groups (*P* = 0.081), suggesting that factors beyond chest wall trauma—such as institutional protocols for drain removal—may influence this outcome. Preserving chest wall integrity with single-port techniques may facilitate earlier mobilization and discharge despite similar drainage durations (24). Postoperative complication rates did not differ significantly between the two approaches (*P* = 0.055), indicating comparable safety.

### Patient satisfaction

Patient-reported satisfaction was assessed in only three studies, with extremely high heterogeneity (I^2^ = 96.4%). The pooled analysis showed no statistically significant difference between the two groups (SMD = 1.480, 95% CI: −0.096 to 3.057, *P* = 0.066). Due to the limited number of studies and substantial heterogeneity, this finding is inconclusive and requires confirmation in future research. The wide confidence interval and high heterogeneity suggest that satisfaction may be influenced by unmeasured factors such as preoperative counseling, scar location, and cultural preferences (25). Future studies employing standardized satisfaction instruments are needed to clarify this potential benefit.

### Surgical efficiency and cost-effectiveness

No significant differences were observed in operative time between single-port and multi-port VATS (MD = 0.134, 95% CI: −0.290 to 0.558, *P* = 0.536). Subgroup analysis revealed that modified single-port techniques tended to have shorter operative times than conventional single-port approaches, although the subgroup difference was not statistically significant. The high heterogeneity in this analysis (I^2^ = 90.2%) likely reflects variations in surgeon experience, institutional volume, and the specific modified techniques employed.

Similarly, the number of staplers used during bullectomy did not differ significantly between groups (MD = 0.132, *P* = 0.815), indicating that the single-port approach does not incur additional consumable costs. This finding is consistent with previous health economic analyses suggesting that single-port VATS is not associated with increased procedural expenditure (26).

No significant difference in postoperative recurrence rate was observed between single-port and multi-port VATS (pooled RR = 0.76, 95% CI: 0.44–1.31, *P* = 0.323), with low heterogeneity across studies (I^2^ = 0.0%). This suggests that the number of incisions is not a determinant of long-term recurrence risk. All included studies employed linear stapler wedge resection combined with mechanical pleurodesis, ensuring adequate bulla excision and pleural symphysis. The core determinants of recurrence—residual apical bullae and incomplete pleurodesis—appear to be equally well addressed by both approaches, consistent with the findings of Yoshikawa et al. (20)

### Heterogeneity sources: conventional vs. modified single-port VATS

Subgroup analysis showed lower heterogeneity in the modified single-port vs. conventional single-port VATS groups. Conventional single-port surgery involves a shared incision for the thoracoscope and instruments, causing frequent collisions and interruptions. Tsuboshima (3) reported that 2–0 nylon suture traction of the pleura reduces instrument collisions from 15–20 to 5–8 per case, eliminating differences in operative time versus triple-port. Song (9) used ENDO KEEPERTM, which fixes thoracoscope/instrument positions via elastic material: this increases operating angle by 15°–20°, expands stapler motion range by 30%, and eliminates time differences vs. triple-port. Additionally, Song's flexible silicone protector cushions nerve compression, and Tsuboshima's chest wall pulley (third intercostal, midaxillary line) reduces suture injury risk by 60%. These modified techniques are not clinically equivalent, and such technical heterogeneity may introduce bias into the pooled results.

### Learning curve of single-port VATS

Kang et al. (8) found single-port VATS took longer than triple-port for less experienced surgeons but not for experienced ones (MD = 1.20 min, *P* = 0.58), indicating a ∼30-case learning curve. Included studies varied in surgeons’ annual caseloads (5–100 cases/year), with inexperienced surgeons prolonging time due to poor instrument coordination. Institutions implementing single-port VATS should provide targeted training for less experienced surgeons (27).

### Limitations and future directions

Several important limitations of this meta-analysis must be acknowledged. First, all 13 included studies were observational cohort studies; the absence of randomized controlled trials introduces inherent risks of selection bias and unmeasured confounding. Second, substantial heterogeneity was observed for several key outcomes, including operative time (I^2^ = 90.2%), postoperative pain (I^2^ = 88.4%), and patient satisfaction (I^2^ = 96.4%), which likely stems from variations in surgical technique, surgeon experience, pleurodesis methods, and outcome measurement tools across institutions. Third, the pooled analyses revealed no statistically significant differences in chest tube duration, patient satisfaction, or postoperative complications. Consequently, the advantages of single-port VATS should not be overstated and appear to be limited to specific domains of recovery and nerve preservation. Fourth, most studies had follow-up durations of less than one year, which is insufficient to assess long-term recurrence rates or chronic post-surgical pain. Therefore, conclusions regarding the procedure's durability and late neurological sequelae remain tentative. Future studies with at least two years of follow-up are needed to confirm the long-term efficacy of single-port VATS. Fifth, two studies did not report standard deviations for certain continuous outcomes, potentially affecting the precision of effect size estimates.

Future directions: Multi-center randomized controlled trials with standardized surgical protocols and core outcome sets are needed to confirm these findings and reduce heterogeneity. Long-term follow-up studies are required to evaluate the durability of recurrence prevention and the trajectory of intercostal nerve recovery.

## Conclusion

In summary, this meta-analysis of available observational data indicates that single-port VATS is associated with a reduced length of stay, reduced postoperative pain, and a lower incidence of chronic chest wall paresthesia than multi-port VATS. The procedure appears non-inferior regarding operative efficiency, complication rates, and recurrence risk. However, given the inherent limitations of the included cohort studies and the observed heterogeneity in secondary outcomes, single-port VATS should be viewed as a valuable and safe alternative rather than the mandatory preferred approach. High-quality randomized controlled trials are needed to establish definitive superiority.

## Data Availability

The data supporting this study are available from the corresponding author upon reasonable request.
