# Supplementary material for: Clinical efficacy and safety of different video-assisted thoracoscopic surgery approaches for bullous lung resection: a systematic review and meta-analysis
Source: Front Surg. 2026 May 29;13:1838672. doi: 10.3389/fsurg.2026.1838672 (PMC13260138; doi:10.3389/fsurg.2026.1838672)
Supplement: Supplementary file 1 [file Table1.docx]

Table S1 Detailed search results of Pubmed database

| Search number | Query | Search Details | Results |
| --- | --- | --- | --- |
| 18 | (((((((((((((((((((Thoracoscopies[Title/Abstract]) OR (Endoscopy, Pleural[Title/Abstract])) OR (Endoscopies, Pleural[Title/Abstract])) OR (Pleural Endoscopies[Title/Abstract])) OR (Pleural Endoscopy[Title/Abstract])) OR (Pleuroscopy[Title/Abstract])) OR (Pleuroscopies[Title/Abstract])) OR (Surgical Procedures, Thoracoscopic[Title/Abstract])) OR (Surgical Procedure, Thoracoscopic[Title/Abstract])) OR (Thoracoscopic Surgical Procedure[Title/Abstract])) OR (Surgery, Thoracoscopic[Title/Abstract])) OR (Surgeries, Thoracoscopic[Title/Abstract])) OR (Thoracoscopic Surgeries[Title/Abstract])) OR (Thoracoscopic Surgery[Title/Abstract])) OR (Thoracoscopic Surgical Procedures[Title/Abstract])) OR ("Thoracoscopy"[Mesh])) ) OR (Thoracoscopy[Title/Abstract])) AND (((((((Bullous Lung[Title/Abstract]) OR (Bullous Emphysema[Title/Abstract])) OR ((Lung[Title/Abstract]) AND (Bullae[Title/Abstract]))) OR ((Lung[Title/Abstract]) AND (Bulla[Title/Abstract]))) OR ((Pulmonary[Title/Abstract]) AND (Bullae[Title/Abstract]))) OR ((Pulmonary[Title/Abstract]) AND (Bulla[Title/Abstract]))) OR ("Pulmonary Bullae Causing Pneumothorax" [Supplementary Concept]))) OR ((((((((((((((((((((Thoracic Surgery, Video-Assisted[Title/Abstract]) OR (Surgeries, Video-Assisted Thoracic[Title/Abstract])) OR (Surgery, Video-Assisted Thoracic[Title/Abstract])) OR (Thoracic Surgeries, Video-Assisted[Title/Abstract])) OR (Thoracic Surgery, Video Assisted[Title/Abstract])) OR (Video-Assisted Thoracic Surgeries[Title/Abstract])) OR (Surgery, Thoracic, Video-Assisted[Title/Abstract])) OR (VATS[Title/Abstract])) OR (VATSs[Title/Abstract])) OR (Video-Assisted Thoracic Surgery[Title/Abstract])) OR (Video Assisted Thoracic Surgery[Title/Abstract])) OR (Video-Assisted Thoracoscopic Surgery[Title/Abstract])) OR (Surgeries, Video-Assisted Thoracoscopic[Title/Abstract])) OR (Surgery, Video-Assisted Thoracoscopic[Title/Abstract])) OR (Thoracoscopic Surgeries, Video-Assisted[Title/Abstract])) OR (Thoracoscopic Surgery, Video-Assisted[Title/Abstract])) OR (Video-Assisted Thoracoscopic Surgeries[Title/Abstract])) OR (Video Assisted Thoracoscopic Surgery[Title/Abstract])) OR ("Thoracic Surgery, Video-Assisted"[Mesh])) AND (((((((Bullous Lung[Title/Abstract]) OR (Bullous Emphysema[Title/Abstract])) OR ((Lung[Title/Abstract]) AND (Bullae[Title/Abstract]))) OR ((Lung[Title/Abstract]) AND (Bulla[Title/Abstract]))) OR ((Pulmonary[Title/Abstract]) AND (Bullae[Title/Abstract]))) OR ((Pulmonary[Title/Abstract]) AND (Bulla[Title/Abstract]))) OR ("Pulmonary Bullae Causing Pneumothorax" [Supplementary Concept]))) | (("Thoracoscopies"[Title/Abstract] OR "endoscopy pleural"[Title/Abstract] OR (("endoscopie"[All Fields] OR "Endoscopy"[MeSH Terms] OR "Endoscopy"[All Fields] OR "Endoscopies"[All Fields] OR "endoscopy s"[All Fields]) AND "Pleural"[Title/Abstract]) OR "pleural endoscopies"[Title/Abstract] OR "pleural endoscopy"[Title/Abstract] OR "Pleuroscopy"[Title/Abstract] OR "Pleuroscopies"[Title/Abstract] OR (("surgical procedures, operative"[MeSH Terms] OR ("Surgical"[All Fields] AND "Procedures"[All Fields] AND "operative"[All Fields]) OR "operative surgical procedures"[All Fields] OR ("Surgical"[All Fields] AND "Procedures"[All Fields]) OR "surgical procedures"[All Fields]) AND "Thoracoscopic"[Title/Abstract]) OR "surgical procedure thoracoscopic"[Title/Abstract] OR "thoracoscopic surgical procedure"[Title/Abstract] OR "surgery thoracoscopic"[Title/Abstract] OR (("Surgery"[MeSH Subheading] OR "Surgery"[All Fields] OR "surgical procedures, operative"[MeSH Terms] OR ("Surgical"[All Fields] AND "Procedures"[All Fields] AND "operative"[All Fields]) OR "operative surgical procedures"[All Fields] OR "general surgery"[MeSH Terms] OR ("general"[All Fields] AND "Surgery"[All Fields]) OR "general surgery"[All Fields] OR "surgery s"[All Fields] OR "surgerys"[All Fields] OR "Surgeries"[All Fields]) AND "Thoracoscopic"[Title/Abstract]) OR "thoracoscopic surgeries"[Title/Abstract] OR "thoracoscopic surgery"[Title/Abstract] OR "thoracoscopic surgical procedures"[Title/Abstract] OR "Thoracoscopy"[MeSH Terms] OR "Thoracoscopy"[Title/Abstract]) AND ("bullous lung"[Title/Abstract] OR "bullous emphysema"[Title/Abstract] OR ("Lung"[Title/Abstract] AND "Bullae"[Title/Abstract]) OR ("Lung"[Title/Abstract] AND "Bulla"[Title/Abstract]) OR ("Pulmonary"[Title/Abstract] AND "Bullae"[Title/Abstract]) OR ("Pulmonary"[Title/Abstract] AND "Bulla"[Title/Abstract]) OR "Pulmonary Bullae Causing Pneumothorax"[Supplementary Concept])) OR (("thoracic surgery video assisted"[Title/Abstract] OR "surgeries video assisted thoracic"[Title/Abstract] OR "surgery video assisted thoracic"[Title/Abstract] OR (("thoracal"[All Fields] OR "thoracical"[All Fields] OR "thorax"[MeSH Terms] OR "thorax"[All Fields] OR "Thoracic"[All Fields] OR "thoracics"[All Fields]) AND "surgeries video assisted"[Title/Abstract]) OR "thoracic surgery video assisted"[Title/Abstract] OR "video assisted thoracic surgeries"[Title/Abstract] OR (("thoracic surgery"[MeSH Terms] OR ("Thoracic"[All Fields] AND "Surgery"[All Fields]) OR "thoracic surgery"[All Fields] OR ("Surgery"[All Fields] AND "Thoracic"[All Fields]) OR "surgery, thoracic"[All Fields]) AND "Video-Assisted"[Title/Abstract]) OR "VATS"[Title/Abstract] OR "VATSs"[Title/Abstract] OR "video assisted thoracic surgery"[Title/Abstract] OR "video assisted thoracic surgery"[Title/Abstract] OR "video assisted thoracoscopic surgery"[Title/Abstract] OR (("Surgery"[MeSH Subheading] OR "Surgery"[All Fields] OR "surgical procedures, operative"[MeSH Terms] OR ("Surgical"[All Fields] AND "Procedures"[All Fields] AND "operative"[All Fields]) OR "operative surgical procedures"[All Fields] OR "general surgery"[MeSH Terms] OR ("general"[All Fields] AND "Surgery"[All Fields]) OR "general surgery"[All Fields] OR "surgery s"[All Fields] OR "surgerys"[All Fields] OR "Surgeries"[All Fields]) AND "video assisted thoracoscopic"[Title/Abstract]) OR "surgery video assisted thoracoscopic"[Title/Abstract] OR "thoracoscopic surgeries video assisted"[Title/Abstract] OR "thoracoscopic surgery video assisted"[Title/Abstract] OR "video assisted thoracoscopic surgeries"[Title/Abstract] OR "video assisted thoracoscopic surgery"[Title/Abstract] OR "thoracic surgery, video assisted"[MeSH Terms]) AND ("bullous lung"[Title/Abstract] OR "bullous emphysema"[Title/Abstract] OR ("Lung"[Title/Abstract] AND "Bullae"[Title/Abstract]) OR ("Lung"[Title/Abstract] AND "Bulla"[Title/Abstract]) OR ("Pulmonary"[Title/Abstract] AND "Bullae"[Title/Abstract]) OR ("Pulmonary"[Title/Abstract] AND "Bulla"[Title/Abstract]) OR "Pulmonary Bullae Causing Pneumothorax"[Supplementary Concept])) | 503 |
| 17 | ((((((((((((((((((Thoracoscopies[Title/Abstract]) OR (Endoscopy, Pleural[Title/Abstract])) OR (Endoscopies, Pleural[Title/Abstract])) OR (Pleural Endoscopies[Title/Abstract])) OR (Pleural Endoscopy[Title/Abstract])) OR (Pleuroscopy[Title/Abstract])) OR (Pleuroscopies[Title/Abstract])) OR (Surgical Procedures, Thoracoscopic[Title/Abstract])) OR (Surgical Procedure, Thoracoscopic[Title/Abstract])) OR (Thoracoscopic Surgical Procedure[Title/Abstract])) OR (Surgery, Thoracoscopic[Title/Abstract])) OR (Surgeries, Thoracoscopic[Title/Abstract])) OR (Thoracoscopic Surgeries[Title/Abstract])) OR (Thoracoscopic Surgery[Title/Abstract])) OR (Thoracoscopic Surgical Procedures[Title/Abstract])) OR ("Thoracoscopy"[Mesh])) ) OR (Thoracoscopy[Title/Abstract])) AND (((((((Bullous Lung[Title/Abstract]) OR (Bullous Emphysema[Title/Abstract])) OR ((Lung[Title/Abstract]) AND (Bullae[Title/Abstract]))) OR ((Lung[Title/Abstract]) AND (Bulla[Title/Abstract]))) OR ((Pulmonary[Title/Abstract]) AND (Bullae[Title/Abstract]))) OR ((Pulmonary[Title/Abstract]) AND (Bulla[Title/Abstract]))) OR ("Pulmonary Bullae Causing Pneumothorax" [Supplementary Concept])) | ("Thoracoscopies"[Title/Abstract] OR "endoscopy pleural"[Title/Abstract] OR (("endoscopie"[All Fields] OR "Endoscopy"[MeSH Terms] OR "Endoscopy"[All Fields] OR "Endoscopies"[All Fields] OR "endoscopy s"[All Fields]) AND "Pleural"[Title/Abstract]) OR "pleural endoscopies"[Title/Abstract] OR "pleural endoscopy"[Title/Abstract] OR "Pleuroscopy"[Title/Abstract] OR "Pleuroscopies"[Title/Abstract] OR (("surgical procedures, operative"[MeSH Terms] OR ("Surgical"[All Fields] AND "Procedures"[All Fields] AND "operative"[All Fields]) OR "operative surgical procedures"[All Fields] OR ("Surgical"[All Fields] AND "Procedures"[All Fields]) OR "surgical procedures"[All Fields]) AND "Thoracoscopic"[Title/Abstract]) OR "surgical procedure thoracoscopic"[Title/Abstract] OR "thoracoscopic surgical procedure"[Title/Abstract] OR "surgery thoracoscopic"[Title/Abstract] OR (("Surgery"[MeSH Subheading] OR "Surgery"[All Fields] OR "surgical procedures, operative"[MeSH Terms] OR ("Surgical"[All Fields] AND "Procedures"[All Fields] AND "operative"[All Fields]) OR "operative surgical procedures"[All Fields] OR "general surgery"[MeSH Terms] OR ("general"[All Fields] AND "Surgery"[All Fields]) OR "general surgery"[All Fields] OR "surgery s"[All Fields] OR "surgerys"[All Fields] OR "Surgeries"[All Fields]) AND "Thoracoscopic"[Title/Abstract]) OR "thoracoscopic surgeries"[Title/Abstract] OR "thoracoscopic surgery"[Title/Abstract] OR "thoracoscopic surgical procedures"[Title/Abstract] OR "Thoracoscopy"[MeSH Terms] OR "Thoracoscopy"[Title/Abstract]) AND ("bullous lung"[Title/Abstract] OR "bullous emphysema"[Title/Abstract] OR ("Lung"[Title/Abstract] AND "Bullae"[Title/Abstract]) OR ("Lung"[Title/Abstract] AND "Bulla"[Title/Abstract]) OR ("Pulmonary"[Title/Abstract] AND "Bullae"[Title/Abstract]) OR ("Pulmonary"[Title/Abstract] AND "Bulla"[Title/Abstract]) OR "Pulmonary Bullae Causing Pneumothorax"[Supplementary Concept]) | 477 |
| 16 | (((((((((((((((((((Thoracic Surgery, Video-Assisted[Title/Abstract]) OR (Surgeries, Video-Assisted Thoracic[Title/Abstract])) OR (Surgery, Video-Assisted Thoracic[Title/Abstract])) OR (Thoracic Surgeries, Video-Assisted[Title/Abstract])) OR (Thoracic Surgery, Video Assisted[Title/Abstract])) OR (Video-Assisted Thoracic Surgeries[Title/Abstract])) OR (Surgery, Thoracic, Video-Assisted[Title/Abstract])) OR (VATS[Title/Abstract])) OR (VATSs[Title/Abstract])) OR (Video-Assisted Thoracic Surgery[Title/Abstract])) OR (Video Assisted Thoracic Surgery[Title/Abstract])) OR (Video-Assisted Thoracoscopic Surgery[Title/Abstract])) OR (Surgeries, Video-Assisted Thoracoscopic[Title/Abstract])) OR (Surgery, Video-Assisted Thoracoscopic[Title/Abstract])) OR (Thoracoscopic Surgeries, Video-Assisted[Title/Abstract])) OR (Thoracoscopic Surgery, Video-Assisted[Title/Abstract])) OR (Video-Assisted Thoracoscopic Surgeries[Title/Abstract])) OR (Video Assisted Thoracoscopic Surgery[Title/Abstract])) OR ("Thoracic Surgery, Video-Assisted"[Mesh])) AND (((((((Bullous Lung[Title/Abstract]) OR (Bullous Emphysema[Title/Abstract])) OR ((Lung[Title/Abstract]) AND (Bullae[Title/Abstract]))) OR ((Lung[Title/Abstract]) AND (Bulla[Title/Abstract]))) OR ((Pulmonary[Title/Abstract]) AND (Bullae[Title/Abstract]))) OR ((Pulmonary[Title/Abstract]) AND (Bulla[Title/Abstract]))) OR ("Pulmonary Bullae Causing Pneumothorax" [Supplementary Concept])) | ("thoracic surgery video assisted"[Title/Abstract] OR "surgeries video assisted thoracic"[Title/Abstract] OR "surgery video assisted thoracic"[Title/Abstract] OR (("thoracal"[All Fields] OR "thoracical"[All Fields] OR "thorax"[MeSH Terms] OR "thorax"[All Fields] OR "Thoracic"[All Fields] OR "thoracics"[All Fields]) AND "surgeries video assisted"[Title/Abstract]) OR "thoracic surgery video assisted"[Title/Abstract] OR "video assisted thoracic surgeries"[Title/Abstract] OR (("thoracic surgery"[MeSH Terms] OR ("Thoracic"[All Fields] AND "Surgery"[All Fields]) OR "thoracic surgery"[All Fields] OR ("Surgery"[All Fields] AND "Thoracic"[All Fields]) OR "surgery, thoracic"[All Fields]) AND "Video-Assisted"[Title/Abstract]) OR "VATS"[Title/Abstract] OR "VATSs"[Title/Abstract] OR "video assisted thoracic surgery"[Title/Abstract] OR "video assisted thoracic surgery"[Title/Abstract] OR "video assisted thoracoscopic surgery"[Title/Abstract] OR (("Surgery"[MeSH Subheading] OR "Surgery"[All Fields] OR "surgical procedures, operative"[MeSH Terms] OR ("surgical"[All Fields] AND "procedures"[All Fields] AND "operative"[All Fields]) OR "operative surgical procedures"[All Fields] OR "general surgery"[MeSH Terms] OR ("general"[All Fields] AND "Surgery"[All Fields]) OR "general surgery"[All Fields] OR "surgery s"[All Fields] OR "surgerys"[All Fields] OR "Surgeries"[All Fields]) AND "video assisted thoracoscopic"[Title/Abstract]) OR "surgery video assisted thoracoscopic"[Title/Abstract] OR "thoracoscopic surgeries video assisted"[Title/Abstract] OR "thoracoscopic surgery video assisted"[Title/Abstract] OR "video assisted thoracoscopic surgeries"[Title/Abstract] OR "video assisted thoracoscopic surgery"[Title/Abstract] OR "thoracic surgery, video assisted"[MeSH Terms]) AND ("bullous lung"[Title/Abstract] OR "bullous emphysema"[Title/Abstract] OR ("Lung"[Title/Abstract] AND "Bullae"[Title/Abstract]) OR ("Lung"[Title/Abstract] AND "Bulla"[Title/Abstract]) OR ("Pulmonary"[Title/Abstract] AND "Bullae"[Title/Abstract]) OR ("Pulmonary"[Title/Abstract] AND "Bulla"[Title/Abstract]) OR "Pulmonary Bullae Causing Pneumothorax"[Supplementary Concept]) | 305 |
| 15 | (((((((((((((((((((Thoracoscopies[Title/Abstract]) OR (Endoscopy, Pleural[Title/Abstract])) OR (Endoscopies, Pleural[Title/Abstract])) OR (Pleural Endoscopies[Title/Abstract])) OR (Pleural Endoscopy[Title/Abstract])) OR (Pleuroscopy[Title/Abstract])) OR (Pleuroscopies[Title/Abstract])) OR (Surgical Procedures, Thoracoscopic[Title/Abstract])) OR (Surgical Procedure, Thoracoscopic[Title/Abstract])) OR (Thoracoscopic Surgical Procedure[Title/Abstract])) OR (Surgery, Thoracoscopic[Title/Abstract])) OR (Surgeries, Thoracoscopic[Title/Abstract])) OR (Thoracoscopic Surgeries[Title/Abstract])) OR (Thoracoscopic Surgery[Title/Abstract])) OR (Thoracoscopic Surgical Procedures[Title/Abstract])) OR ("Thoracoscopy"[Mesh])) ) OR (Thoracoscopy[Title/Abstract])) AND (((((((((((((((((((Thoracic Surgery, Video-Assisted[Title/Abstract]) OR (Surgeries, Video-Assisted Thoracic[Title/Abstract])) OR (Surgery, Video-Assisted Thoracic[Title/Abstract])) OR (Thoracic Surgeries, Video-Assisted[Title/Abstract])) OR (Thoracic Surgery, Video Assisted[Title/Abstract])) OR (Video-Assisted Thoracic Surgeries[Title/Abstract])) OR (Surgery, Thoracic, Video-Assisted[Title/Abstract])) OR (VATS[Title/Abstract])) OR (VATSs[Title/Abstract])) OR (Video-Assisted Thoracic Surgery[Title/Abstract])) OR (Video Assisted Thoracic Surgery[Title/Abstract])) OR (Video-Assisted Thoracoscopic Surgery[Title/Abstract])) OR (Surgeries, Video-Assisted Thoracoscopic[Title/Abstract])) OR (Surgery, Video-Assisted Thoracoscopic[Title/Abstract])) OR (Thoracoscopic Surgeries, Video-Assisted[Title/Abstract])) OR (Thoracoscopic Surgery, Video-Assisted[Title/Abstract])) OR (Video-Assisted Thoracoscopic Surgeries[Title/Abstract])) OR (Video Assisted Thoracoscopic Surgery[Title/Abstract])) OR ("Thoracic Surgery, Video-Assisted"[Mesh]))) AND (((((((Bullous Lung[Title/Abstract]) OR (Bullous Emphysema[Title/Abstract])) OR ((Lung[Title/Abstract]) AND (Bullae[Title/Abstract]))) OR ((Lung[Title/Abstract]) AND (Bulla[Title/Abstract]))) OR ((Pulmonary[Title/Abstract]) AND (Bullae[Title/Abstract]))) OR ((Pulmonary[Title/Abstract]) AND (Bulla[Title/Abstract]))) OR ("Pulmonary Bullae Causing Pneumothorax" [Supplementary Concept])) | ("Thoracoscopies"[Title/Abstract] OR "endoscopy pleural"[Title/Abstract] OR (("endoscopie"[All Fields] OR "Endoscopy"[MeSH Terms] OR "Endoscopy"[All Fields] OR "Endoscopies"[All Fields] OR "endoscopy s"[All Fields]) AND "Pleural"[Title/Abstract]) OR "pleural endoscopies"[Title/Abstract] OR "pleural endoscopy"[Title/Abstract] OR "Pleuroscopy"[Title/Abstract] OR "Pleuroscopies"[Title/Abstract] OR (("surgical procedures, operative"[MeSH Terms] OR ("Surgical"[All Fields] AND "Procedures"[All Fields] AND "operative"[All Fields]) OR "operative surgical procedures"[All Fields] OR ("Surgical"[All Fields] AND "Procedures"[All Fields]) OR "surgical procedures"[All Fields]) AND "Thoracoscopic"[Title/Abstract]) OR "surgical procedure thoracoscopic"[Title/Abstract] OR "thoracoscopic surgical procedure"[Title/Abstract] OR "surgery thoracoscopic"[Title/Abstract] OR (("Surgery"[MeSH Subheading] OR "Surgery"[All Fields] OR "surgical procedures, operative"[MeSH Terms] OR ("Surgical"[All Fields] AND "Procedures"[All Fields] AND "operative"[All Fields]) OR "operative surgical procedures"[All Fields] OR "general surgery"[MeSH Terms] OR ("general"[All Fields] AND "Surgery"[All Fields]) OR "general surgery"[All Fields] OR "surgery s"[All Fields] OR "surgerys"[All Fields] OR "Surgeries"[All Fields]) AND "Thoracoscopic"[Title/Abstract]) OR "thoracoscopic surgeries"[Title/Abstract] OR "thoracoscopic surgery"[Title/Abstract] OR "thoracoscopic surgical procedures"[Title/Abstract] OR "Thoracoscopy"[MeSH Terms] OR "Thoracoscopy"[Title/Abstract]) AND ("thoracic surgery video assisted"[Title/Abstract] OR "surgeries video assisted thoracic"[Title/Abstract] OR "surgery video assisted thoracic"[Title/Abstract] OR (("thoracal"[All Fields] OR "thoracical"[All Fields] OR "thorax"[MeSH Terms] OR "thorax"[All Fields] OR "Thoracic"[All Fields] OR "thoracics"[All Fields]) AND "surgeries video assisted"[Title/Abstract]) OR "thoracic surgery video assisted"[Title/Abstract] OR "video assisted thoracic surgeries"[Title/Abstract] OR (("thoracic surgery"[MeSH Terms] OR ("Thoracic"[All Fields] AND "Surgery"[All Fields]) OR "thoracic surgery"[All Fields] OR ("Surgery"[All Fields] AND "Thoracic"[All Fields]) OR "surgery, thoracic"[All Fields]) AND "Video-Assisted"[Title/Abstract]) OR "VATS"[Title/Abstract] OR "VATSs"[Title/Abstract] OR "video assisted thoracic surgery"[Title/Abstract] OR "video assisted thoracic surgery"[Title/Abstract] OR "video assisted thoracoscopic surgery"[Title/Abstract] OR (("Surgery"[MeSH Subheading] OR "Surgery"[All Fields] OR "surgical procedures, operative"[MeSH Terms] OR ("Surgical"[All Fields] AND "Procedures"[All Fields] AND "operative"[All Fields]) OR "operative surgical procedures"[All Fields] OR "general surgery"[MeSH Terms] OR ("general"[All Fields] AND "Surgery"[All Fields]) OR "general surgery"[All Fields] OR "surgery s"[All Fields] OR "surgerys"[All Fields] OR "Surgeries"[All Fields]) AND "video assisted thoracoscopic"[Title/Abstract]) OR "surgery video assisted thoracoscopic"[Title/Abstract] OR "thoracoscopic surgeries video assisted"[Title/Abstract] OR "thoracoscopic surgery video assisted"[Title/Abstract] OR "video assisted thoracoscopic surgeries"[Title/Abstract] OR "video assisted thoracoscopic surgery"[Title/Abstract] OR "thoracic surgery, video assisted"[MeSH Terms]) AND ("bullous lung"[Title/Abstract] OR "bullous emphysema"[Title/Abstract] OR ("Lung"[Title/Abstract] AND "Bullae"[Title/Abstract]) OR ("Lung"[Title/Abstract] AND "Bulla"[Title/Abstract]) OR ("Pulmonary"[Title/Abstract] AND "Bullae"[Title/Abstract]) OR ("Pulmonary"[Title/Abstract] AND "Bulla"[Title/Abstract]) OR "Pulmonary Bullae Causing Pneumothorax"[Supplementary Concept]) | 279 |
| 14 | ((((((((((((((((((Thoracoscopies[Title/Abstract]) OR (Endoscopy, Pleural[Title/Abstract])) OR (Endoscopies, Pleural[Title/Abstract])) OR (Pleural Endoscopies[Title/Abstract])) OR (Pleural Endoscopy[Title/Abstract])) OR (Pleuroscopy[Title/Abstract])) OR (Pleuroscopies[Title/Abstract])) OR (Surgical Procedures, Thoracoscopic[Title/Abstract])) OR (Surgical Procedure, Thoracoscopic[Title/Abstract])) OR (Thoracoscopic Surgical Procedure[Title/Abstract])) OR (Surgery, Thoracoscopic[Title/Abstract])) OR (Surgeries, Thoracoscopic[Title/Abstract])) OR (Thoracoscopic Surgeries[Title/Abstract])) OR (Thoracoscopic Surgery[Title/Abstract])) OR (Thoracoscopic Surgical Procedures[Title/Abstract])) OR ("Thoracoscopy"[Mesh])) ) OR (Thoracoscopy[Title/Abstract])) AND (((((((((((((((((((Thoracic Surgery, Video-Assisted[Title/Abstract]) OR (Surgeries, Video-Assisted Thoracic[Title/Abstract])) OR (Surgery, Video-Assisted Thoracic[Title/Abstract])) OR (Thoracic Surgeries, Video-Assisted[Title/Abstract])) OR (Thoracic Surgery, Video Assisted[Title/Abstract])) OR (Video-Assisted Thoracic Surgeries[Title/Abstract])) OR (Surgery, Thoracic, Video-Assisted[Title/Abstract])) OR (VATS[Title/Abstract])) OR (VATSs[Title/Abstract])) OR (Video-Assisted Thoracic Surgery[Title/Abstract])) OR (Video Assisted Thoracic Surgery[Title/Abstract])) OR (Video-Assisted Thoracoscopic Surgery[Title/Abstract])) OR (Surgeries, Video-Assisted Thoracoscopic[Title/Abstract])) OR (Surgery, Video-Assisted Thoracoscopic[Title/Abstract])) OR (Thoracoscopic Surgeries, Video-Assisted[Title/Abstract])) OR (Thoracoscopic Surgery, Video-Assisted[Title/Abstract])) OR (Video-Assisted Thoracoscopic Surgeries[Title/Abstract])) OR (Video Assisted Thoracoscopic Surgery[Title/Abstract])) OR ("Thoracic Surgery, Video-Assisted"[Mesh])) | ("Thoracoscopies"[Title/Abstract] OR "endoscopy pleural"[Title/Abstract] OR (("endoscopie"[All Fields] OR "Endoscopy"[MeSH Terms] OR "Endoscopy"[All Fields] OR "Endoscopies"[All Fields] OR "endoscopy s"[All Fields]) AND "Pleural"[Title/Abstract]) OR "pleural endoscopies"[Title/Abstract] OR "pleural endoscopy"[Title/Abstract] OR "Pleuroscopy"[Title/Abstract] OR "Pleuroscopies"[Title/Abstract] OR (("surgical procedures, operative"[MeSH Terms] OR ("Surgical"[All Fields] AND "Procedures"[All Fields] AND "operative"[All Fields]) OR "operative surgical procedures"[All Fields] OR ("Surgical"[All Fields] AND "Procedures"[All Fields]) OR "surgical procedures"[All Fields]) AND "Thoracoscopic"[Title/Abstract]) OR "surgical procedure thoracoscopic"[Title/Abstract] OR "thoracoscopic surgical procedure"[Title/Abstract] OR "surgery thoracoscopic"[Title/Abstract] OR (("Surgery"[MeSH Subheading] OR "Surgery"[All Fields] OR "surgical procedures, operative"[MeSH Terms] OR ("Surgical"[All Fields] AND "Procedures"[All Fields] AND "operative"[All Fields]) OR "operative surgical procedures"[All Fields] OR "general surgery"[MeSH Terms] OR ("general"[All Fields] AND "Surgery"[All Fields]) OR "general surgery"[All Fields] OR "surgery s"[All Fields] OR "surgerys"[All Fields] OR "Surgeries"[All Fields]) AND "Thoracoscopic"[Title/Abstract]) OR "thoracoscopic surgeries"[Title/Abstract] OR "thoracoscopic surgery"[Title/Abstract] OR "thoracoscopic surgical procedures"[Title/Abstract] OR "Thoracoscopy"[MeSH Terms] OR "Thoracoscopy"[Title/Abstract]) AND ("thoracic surgery video assisted"[Title/Abstract] OR "surgeries video assisted thoracic"[Title/Abstract] OR "surgery video assisted thoracic"[Title/Abstract] OR (("thoracal"[All Fields] OR "thoracical"[All Fields] OR "thorax"[MeSH Terms] OR "thorax"[All Fields] OR "Thoracic"[All Fields] OR "thoracics"[All Fields]) AND "surgeries video assisted"[Title/Abstract]) OR "thoracic surgery video assisted"[Title/Abstract] OR "video assisted thoracic surgeries"[Title/Abstract] OR (("thoracic surgery"[MeSH Terms] OR ("Thoracic"[All Fields] AND "Surgery"[All Fields]) OR "thoracic surgery"[All Fields] OR ("Surgery"[All Fields] AND "Thoracic"[All Fields]) OR "surgery, thoracic"[All Fields]) AND "Video-Assisted"[Title/Abstract]) OR "VATS"[Title/Abstract] OR "VATSs"[Title/Abstract] OR "video assisted thoracic surgery"[Title/Abstract] OR "video assisted thoracic surgery"[Title/Abstract] OR "video assisted thoracoscopic surgery"[Title/Abstract] OR (("Surgery"[MeSH Subheading] OR "Surgery"[All Fields] OR "surgical procedures, operative"[MeSH Terms] OR ("Surgical"[All Fields] AND "Procedures"[All Fields] AND "operative"[All Fields]) OR "operative surgical procedures"[All Fields] OR "general surgery"[MeSH Terms] OR ("general"[All Fields] AND "Surgery"[All Fields]) OR "general surgery"[All Fields] OR "surgery s"[All Fields] OR "surgerys"[All Fields] OR "Surgeries"[All Fields]) AND "video assisted thoracoscopic"[Title/Abstract]) OR "surgery video assisted thoracoscopic"[Title/Abstract] OR "thoracoscopic surgeries video assisted"[Title/Abstract] OR "thoracoscopic surgery video assisted"[Title/Abstract] OR "video assisted thoracoscopic surgeries"[Title/Abstract] OR "video assisted thoracoscopic surgery"[Title/Abstract] OR "thoracic surgery, video assisted"[MeSH Terms]) | 14,705 |
| 13 | ((((((((((((((((((Thoracic Surgery, Video-Assisted[Title/Abstract]) OR (Surgeries, Video-Assisted Thoracic[Title/Abstract])) OR (Surgery, Video-Assisted Thoracic[Title/Abstract])) OR (Thoracic Surgeries, Video-Assisted[Title/Abstract])) OR (Thoracic Surgery, Video Assisted[Title/Abstract])) OR (Video-Assisted Thoracic Surgeries[Title/Abstract])) OR (Surgery, Thoracic, Video-Assisted[Title/Abstract])) OR (VATS[Title/Abstract])) OR (VATSs[Title/Abstract])) OR (Video-Assisted Thoracic Surgery[Title/Abstract])) OR (Video Assisted Thoracic Surgery[Title/Abstract])) OR (Video-Assisted Thoracoscopic Surgery[Title/Abstract])) OR (Surgeries, Video-Assisted Thoracoscopic[Title/Abstract])) OR (Surgery, Video-Assisted Thoracoscopic[Title/Abstract])) OR (Thoracoscopic Surgeries, Video-Assisted[Title/Abstract])) OR (Thoracoscopic Surgery, Video-Assisted[Title/Abstract])) OR (Video-Assisted Thoracoscopic Surgeries[Title/Abstract])) OR (Video Assisted Thoracoscopic Surgery[Title/Abstract])) OR ("Thoracic Surgery, Video-Assisted"[Mesh]) | "thoracic surgery video assisted"[Title/Abstract] OR "surgeries video assisted thoracic"[Title/Abstract] OR "surgery video assisted thoracic"[Title/Abstract] OR (("thoracal"[All Fields] OR "thoracical"[All Fields] OR "thorax"[MeSH Terms] OR "thorax"[All Fields] OR "Thoracic"[All Fields] OR "thoracics"[All Fields]) AND "surgeries video assisted"[Title/Abstract]) OR "thoracic surgery video assisted"[Title/Abstract] OR "video assisted thoracic surgeries"[Title/Abstract] OR (("thoracic surgery"[MeSH Terms] OR ("Thoracic"[All Fields] AND "Surgery"[All Fields]) OR "thoracic surgery"[All Fields] OR ("Surgery"[All Fields] AND "Thoracic"[All Fields]) OR "surgery, thoracic"[All Fields]) AND "Video-Assisted"[Title/Abstract]) OR "VATS"[Title/Abstract] OR "VATSs"[Title/Abstract] OR "video assisted thoracic surgery"[Title/Abstract] OR "video assisted thoracic surgery"[Title/Abstract] OR "video assisted thoracoscopic surgery"[Title/Abstract] OR (("Surgery"[MeSH Subheading] OR "Surgery"[All Fields] OR "surgical procedures, operative"[MeSH Terms] OR ("surgical"[All Fields] AND "procedures"[All Fields] AND "operative"[All Fields]) OR "operative surgical procedures"[All Fields] OR "general surgery"[MeSH Terms] OR ("general"[All Fields] AND "Surgery"[All Fields]) OR "general surgery"[All Fields] OR "surgery s"[All Fields] OR "surgerys"[All Fields] OR "Surgeries"[All Fields]) AND "video assisted thoracoscopic"[Title/Abstract]) OR "surgery video assisted thoracoscopic"[Title/Abstract] OR "thoracoscopic surgeries video assisted"[Title/Abstract] OR "thoracoscopic surgery video assisted"[Title/Abstract] OR "video assisted thoracoscopic surgeries"[Title/Abstract] OR "video assisted thoracoscopic surgery"[Title/Abstract] OR "thoracic surgery, video assisted"[MeSH Terms] | 17,152 |
| 12 | (((((((((((((((((Thoracoscopies[Title/Abstract]) OR (Endoscopy, Pleural[Title/Abstract])) OR (Endoscopies, Pleural[Title/Abstract])) OR (Pleural Endoscopies[Title/Abstract])) OR (Pleural Endoscopy[Title/Abstract])) OR (Pleuroscopy[Title/Abstract])) OR (Pleuroscopies[Title/Abstract])) OR (Surgical Procedures, Thoracoscopic[Title/Abstract])) OR (Surgical Procedure, Thoracoscopic[Title/Abstract])) OR (Thoracoscopic Surgical Procedure[Title/Abstract])) OR (Surgery, Thoracoscopic[Title/Abstract])) OR (Surgeries, Thoracoscopic[Title/Abstract])) OR (Thoracoscopic Surgeries[Title/Abstract])) OR (Thoracoscopic Surgery[Title/Abstract])) OR (Thoracoscopic Surgical Procedures[Title/Abstract])) OR ("Thoracoscopy"[Mesh])) ) OR (Thoracoscopy[Title/Abstract]) | "Thoracoscopies"[Title/Abstract] OR "endoscopy pleural"[Title/Abstract] OR (("endoscopie"[All Fields] OR "Endoscopy"[MeSH Terms] OR "Endoscopy"[All Fields] OR "Endoscopies"[All Fields] OR "endoscopy s"[All Fields]) AND "Pleural"[Title/Abstract]) OR "pleural endoscopies"[Title/Abstract] OR "pleural endoscopy"[Title/Abstract] OR "Pleuroscopy"[Title/Abstract] OR "Pleuroscopies"[Title/Abstract] OR (("surgical procedures, operative"[MeSH Terms] OR ("Surgical"[All Fields] AND "Procedures"[All Fields] AND "operative"[All Fields]) OR "operative surgical procedures"[All Fields] OR ("Surgical"[All Fields] AND "Procedures"[All Fields]) OR "surgical procedures"[All Fields]) AND "Thoracoscopic"[Title/Abstract]) OR "surgical procedure thoracoscopic"[Title/Abstract] OR "thoracoscopic surgical procedure"[Title/Abstract] OR "surgery thoracoscopic"[Title/Abstract] OR (("Surgery"[MeSH Subheading] OR "Surgery"[All Fields] OR "surgical procedures, operative"[MeSH Terms] OR ("Surgical"[All Fields] AND "Procedures"[All Fields] AND "operative"[All Fields]) OR "operative surgical procedures"[All Fields] OR "general surgery"[MeSH Terms] OR ("general"[All Fields] AND "Surgery"[All Fields]) OR "general surgery"[All Fields] OR "surgery s"[All Fields] OR "surgerys"[All Fields] OR "Surgeries"[All Fields]) AND "Thoracoscopic"[Title/Abstract]) OR "thoracoscopic surgeries"[Title/Abstract] OR "thoracoscopic surgery"[Title/Abstract] OR "thoracoscopic surgical procedures"[Title/Abstract] OR "Thoracoscopy"[MeSH Terms] OR "Thoracoscopy"[Title/Abstract] | 29,911 |
| 10 | "Thoracic Surgery, Video-Assisted"[Mesh] | "thoracic surgery, video assisted"[MeSH Terms] | 9,570 |
| 9 | ((((((Bullous Lung[Title/Abstract]) OR (Bullous Emphysema[Title/Abstract])) OR ((Lung[Title/Abstract]) AND (Bullae[Title/Abstract]))) OR ((Lung[Title/Abstract]) AND (Bulla[Title/Abstract]))) OR ((Pulmonary[Title/Abstract]) AND (Bullae[Title/Abstract]))) OR ((Pulmonary[Title/Abstract]) AND (Bulla[Title/Abstract]))) OR ("Pulmonary Bullae Causing Pneumothorax" [Supplementary Concept]) | "bullous lung"[Title/Abstract] OR "bullous emphysema"[Title/Abstract] OR ("Lung"[Title/Abstract] AND "Bullae"[Title/Abstract]) OR ("Lung"[Title/Abstract] AND "Bulla"[Title/Abstract]) OR ("Pulmonary"[Title/Abstract] AND "Bullae"[Title/Abstract]) OR ("Pulmonary"[Title/Abstract] AND "Bulla"[Title/Abstract]) OR "Pulmonary Bullae Causing Pneumothorax"[Supplementary Concept] | 2,437 |
| 8 | Bullous Lung[Title/Abstract] | "bullous lung"[Title/Abstract] | 197 |
| 7 | Bullous Emphysema[Title/Abstract] | "bullous emphysema"[Title/Abstract] | 577 |
| 6 | (Lung[Title/Abstract]) AND (Bullae[Title/Abstract]) | "Lung"[Title/Abstract] AND "Bullae"[Title/Abstract] | 1,107 |
| 5 | (Lung[Title/Abstract]) AND (Bulla[Title/Abstract]) | "Lung"[Title/Abstract] AND "Bulla"[Title/Abstract] | 561 |
| 4 | (Pulmonary[Title/Abstract]) AND (Bullae[Title/Abstract]) | "Pulmonary"[Title/Abstract] AND "Bullae"[Title/Abstract] | 897 |
| 3 | (Pulmonary[Title/Abstract]) AND (Bulla[Title/Abstract]) | "Pulmonary"[Title/Abstract] AND "Bulla"[Title/Abstract] | 424 |
| 2 | "Pulmonary Bullae Causing Pneumothorax" [Supplementary Concept] | "Pulmonary Bullae Causing Pneumothorax"[Supplementary Concept] | 7 |
| 1 | "Thoracoscopy"[Mesh] | "Thoracoscopy"[MeSH Terms] | 17,831 |

Table S2 Detailed search results of Web of science database

| Num | Search Query | Database | Results |
| --- | --- | --- | --- |
| 1 | bulla (Topic) OR bullae (Topic) and Preprint Citation Index (Exclude – Database) | All Databases | 8515 |
| 2 | Pulmonary (Topic) OR Lung (Topic) and Preprint Citation Index (Exclude – Database) | All Databases | 2657523 |
| 3 | #1 AND #2 and Preprint Citation Index (Exclude – Database) | All Databases | 2653 |
| 4 | Thoracoscopy (Topic) OR Thoracoscopies (Topic) OR Endoscopy, Pleural (Topic) OR Endoscopies, Pleural (Topic) OR Pleural Endoscopies (Topic) OR Pleural Endoscopy (Topic) OR Pleuroscopy (Topic) OR Pleuroscopies (Topic) OR Surgical Procedures, Thoracoscopic (Topic) OR Surgical Procedure, Thoracoscopic (Topic) OR Thoracoscopic Surgical Procedure (Topic) OR Surgery, Thoracoscopic (Topic) OR Surgeries, Thoracoscopic (Topic) OR Thoracoscopic Surgeries (Topic) OR Thoracoscopic Surgery (Topic) OR Thoracoscopic Surgical Procedures (Topic) and Preprint Citation Index (Exclude – Database) | All Databases | 30073 |
| 5 | Mini-QuEChERS (Topic) AND antidepressant drugs (Topic) and Preprint Citation Index (Exclude – Database) | All Databases | 3 |
| 6 | Yangzhou Univ, Coll Int Studies (Address) and Preprint Citation Index (Exclude – Database) | All Databases | 15 |
| 7 | Thoracic Surgery, Video-Assisted (Topic) OR Surgeries, Video-Assisted Thoracic (Topic) OR Surgery, Video-Assisted Thoracic (Topic) OR Thoracic Surgeries, Video-Assisted (Topic) OR Thoracic Surgery, Video Assisted (Topic) OR Video-Assisted Thoracic Surgeries (Topic) OR Surgery, Thoracic, Video-Assisted (Topic) OR VATS (Topic) OR VATSs (Topic) OR Video-Assisted Thoracic Surgery (Topic) OR Video Assisted Thoracic Surgery (Topic) OR Video-Assisted Thoracoscopic Surgery (Topic) OR Surgeries, Video-Assisted Thoracoscopic (Topic) OR Surgery, Video-Assisted Thoracoscopic (Topic) OR Thoracoscopic Surgeries, Video-Assisted (Topic) OR Thoracoscopic Surgery, Video-Assisted (Topic) OR Video-Assisted Thoracoscopic Surgeries (Topic) OR Video Assisted Thoracoscopic Surgery (Topic) and Preprint Citation Index (Exclude – Database) | All Databases | 31945 |
| 8 | Pulmonary Bullae Causing Pneumothorax (Topic) and Preprint Citation Index (Exclude – Database) | All Databases | 190 |
| 9 | #8 OR #3 and Preprint Citation Index (Exclude – Database) | All Databases | 2653 |
| 10 | #4 OR #7 and Preprint Citation Index (Exclude – Database) | All Databases | 48864 |
| 11 | #9 AND #10 and Preprint Citation Index (Exclude – Database) | All Databases | 563 |

Table S3 Detailed search results of Cochrane database

| NUM. | Query | Results |
| --- | --- | --- |
| #1 | Pulmonary bullae | 44 |
| #2 | lung bullae | 62 |
| #3 | Pulmonary Bullae Causing Pneumothorax | 3 |
| #4 | Lung bulla | 21 |
| #5 | Pulmonary bulla | 20 |
| #6 | #1 OR #2 OR #3 OR #4 OR #5 | 87 |
| #7 | MeSH descriptor: [Thoracoscopy] explode all trees | 867 |
| #8 | (Thoracoscopy).it.ab.kw OR (Thoracoscopies).it.ab.kw... | 968 |
| #9 | (Pleural Endoscopy).it.ab.kw OR (Pleuroscopy)... | 438 |
| #10 | (Thoracoscopic Surgical Procedure).it.ab.kw... | 2615 |
| #11 | Thoracoscopic Surgical Procedures | 289 |
| #12 | #7 OR #8 OR #9 OR #10 OR #11 | 3276 |
| #13 | MeSH descriptor: [Thoracic Surgery, Video-Assisted] explode all trees | 586 |
| #14 | (Thoracic Surgery, Video-Assisted).it.ab.kw... | 1296 |
| #15 | (Video-assisted Thoracic Surgeries).it.ab.kw... | 1852 |
| #16 | (Video Assisted Thoracic Surgery).it.ab.kw... | 1772 |
| #17 | (Thoracoscopic Surgery, Video-Assisted).it.ab.kw... | 1431 |
| #18 | #13 OR #14 OR #15 OR #16 OR #17 | 2106 |
| #19 | #12 OR #18 | 3727 |
| #20 | #19 AND #6 | 41 |

Table S4 Detailed search results of Embase database

| No. | Query | Results |
| --- | --- | --- |
| #13 | #11 AND #12 | 688 |
| #12 | #6 OR #9 | 46550 |
| #11 | #3 OR #10 | 4329 |
| #10 | 'pulmonary bullae causing pneumothorax' OR (pulmonary bullae causing AND ('pneumothorax'/exp OR pneumothorax)) | 47 |
| #9 | #7 OR #8 | 28518 |
| #8 | 'video assisted thoracoscopic surgery'/exp OR 'video assisted thoracoscopic surgery' OR (('video'/exp OR video) AND assisted AND thoracoscopic AND ('surgery'/exp OR surgery)) OR 'thoracic surgery, video-assisted':ab,ti OR vats:ab,ti OR 'video assisted thoracic surgery':ab,ti OR 'video assisted thorax surgery':ab,ti OR 'video thoracoscopic surgery':ab,ti OR 'video-assisted thoracic surgery':ab,ti OR ('video-assisted thoracoscopic surgical':ab,ti AND vats:ab,ti AND resection:ab,ti) OR 'video-assisted thoracoscopic surgical approach':ab,ti OR 'video-assisted thoracoscopic surgical resection':ab,ti OR 'video-assisted thorascopic surgery':ab,ti OR 'video-assisted thorascopic surgical resection':ab,ti OR 'videothoracoscopic surgery':ab,ti OR 'video assisted thoracoscopic surgery':ab,ti | 28518 |
| #7 | 'video assisted thoracoscopic surgery'/exp | 20955 |
| #6 | #4 OR #5 | 41548 |
| #5 | 'thoracoscopy'/exp OR thoracoscopy OR 'pleural endoscopy':ab,ti OR thoracoscopy:ab,ti | 41548 |
| #4 | 'thoracoscopy'/exp | 39760 |
| #3 | #1 OR #2 | 4321 |
| #2 | 'lung bulla'/exp OR 'lung bulla' OR (('lung'/exp OR lung) AND ('bulla'/exp OR bulla)) OR 'bulla, lung':ab,ti OR 'bullous lung disease':ab,ti OR 'bullous pulmonary disease':ab,ti OR 'lung bullae':ab,ti OR 'lung bullous disease':ab,ti OR 'pulmonary bulla':ab,ti OR 'pulmonary bullae':ab,ti OR 'pulmonary bullous disease':ab,ti OR 'pulmonary bullous lesion':ab,ti OR 'lung bulla':ab,ti | 4321 |
| #1 | 'lung bulla'/exp | 1510 |
