# Supplementary material for: Clinical efficacy and safety of different video-assisted thoracoscopic surgery approaches for bullous lung resection: a systematic review and meta-analysis
Source: Front Surg. 2026 May 29;13:1838672. doi: 10.3389/fsurg.2026.1838672 (PMC13260138; doi:10.3389/fsurg.2026.1838672)
Supplement: Supplementary file 2 [file Supplementaryfile1.docx]

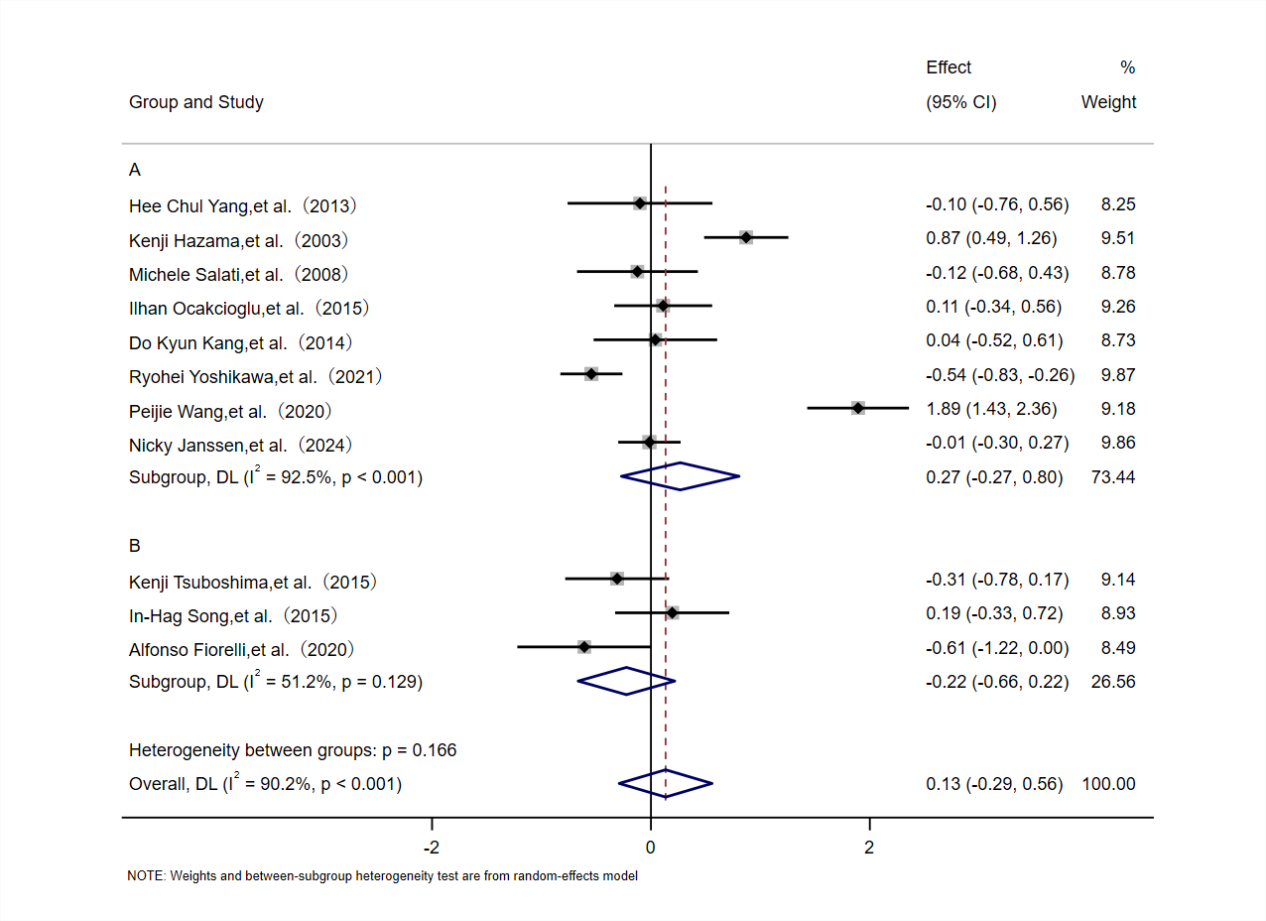


**Supplementary Figure S1 Comparison of operation time between single-hole and three-hole VATS pulmonary bullae resection**

Group A : ordinary single-port VATS group ; group B : Modified uniportal VATS group

**Supplementary Figure S2 Sensitivity analysis of the operation time of single-hole and three-hole VATS pulmonary bulla resection**

**
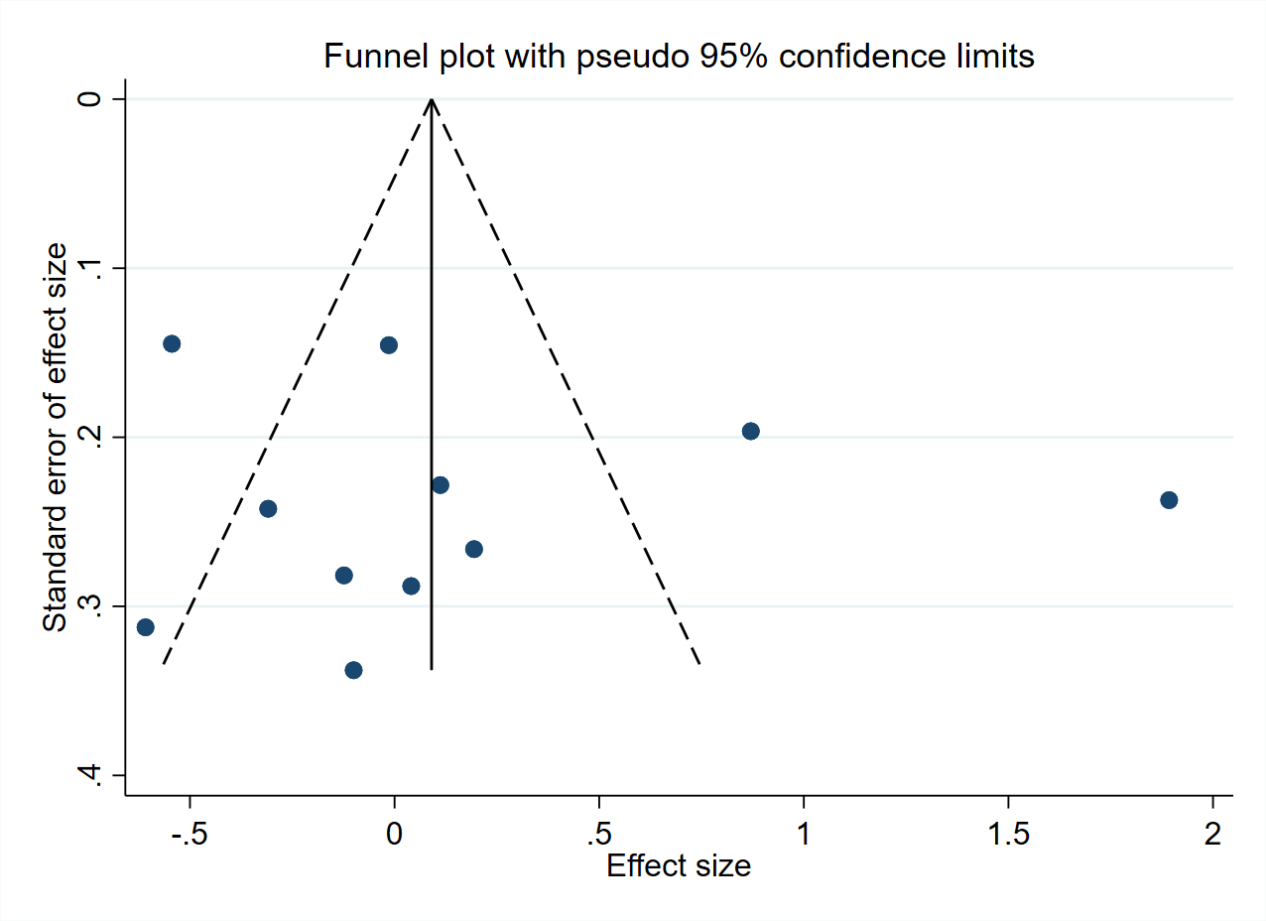
**

**Supplementary Figure S3 Publication bias funnel plot of operation time of single-hole and three-hole VATS pulmonary bullae resection**


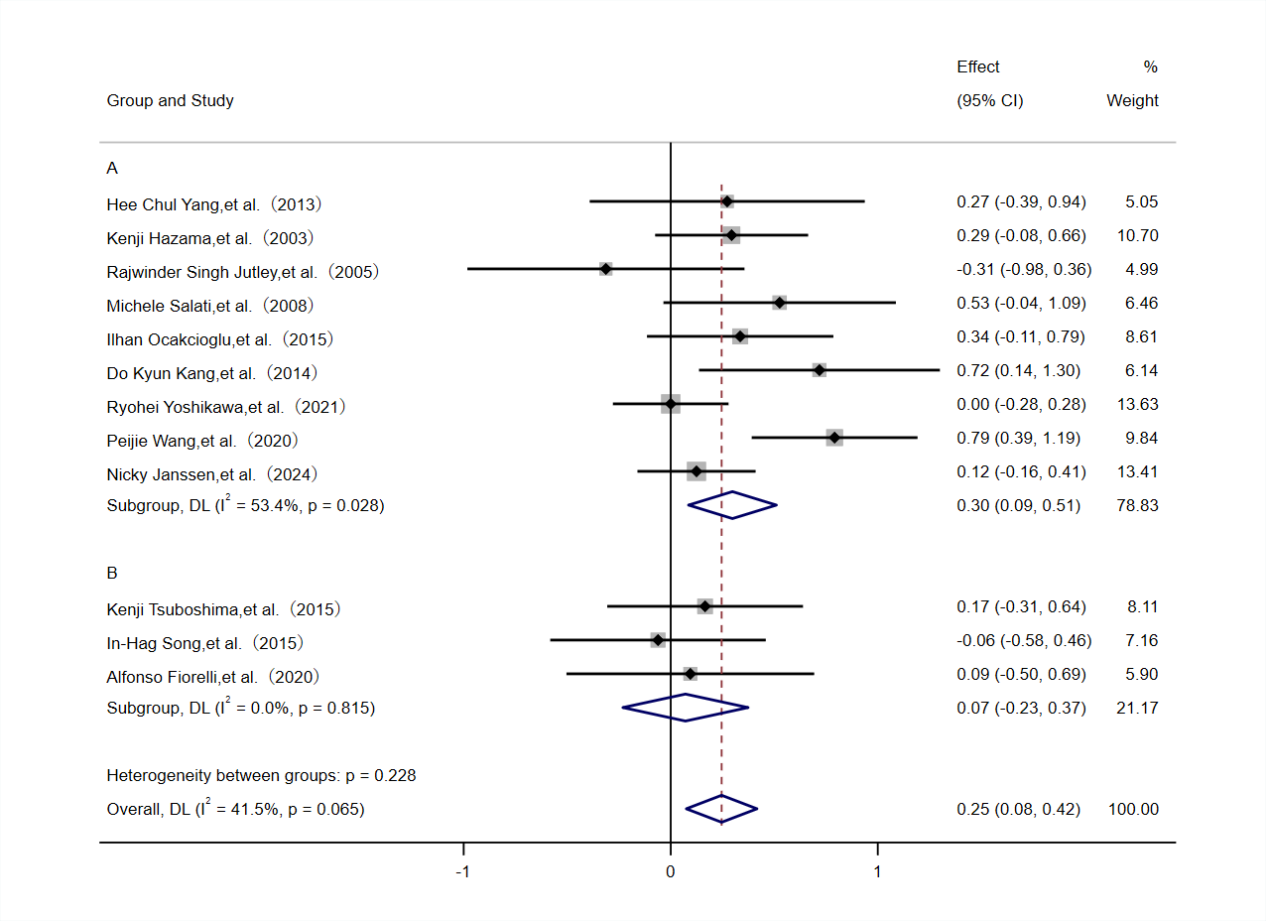


**Supplementary Figure S4 Comparison of hospitalization time between single-hole and three-hole VATS pulmonary bullae resection**

Group A : ordinary single-port VATS group ; group B : Modified uniportal VATS group


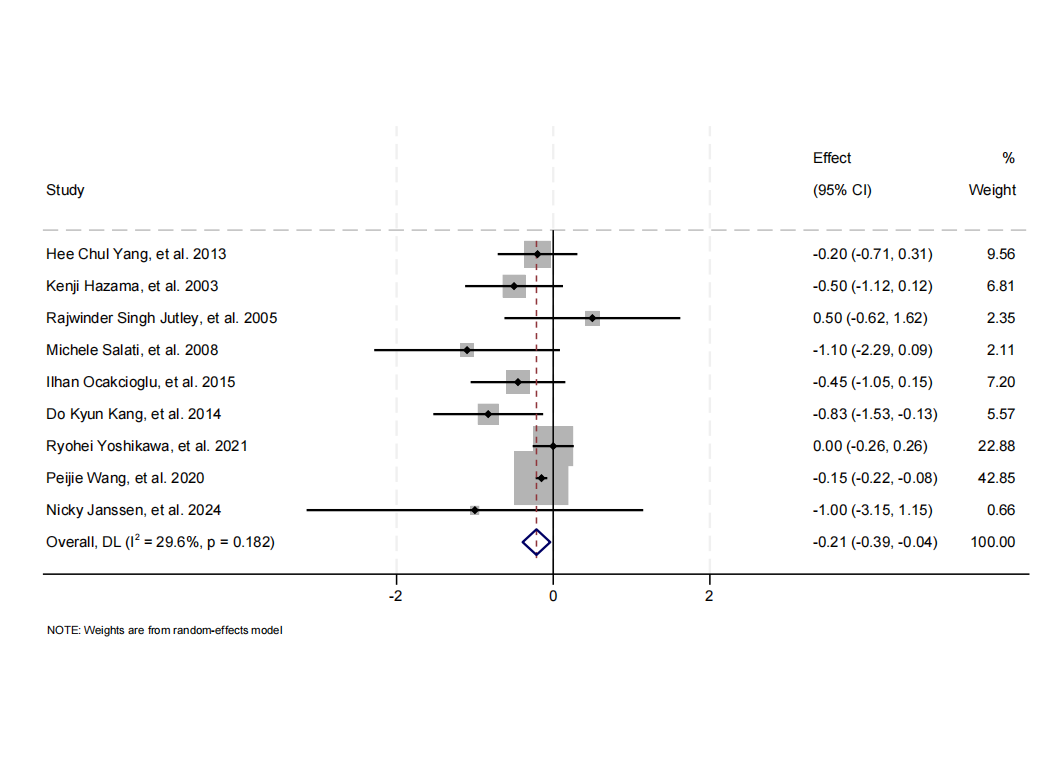


**Supplementary Figure S4a Forest plot of hospital stay comparing conventional single-port versus multi-port VATS (excluding all modified single-port studies)**

**
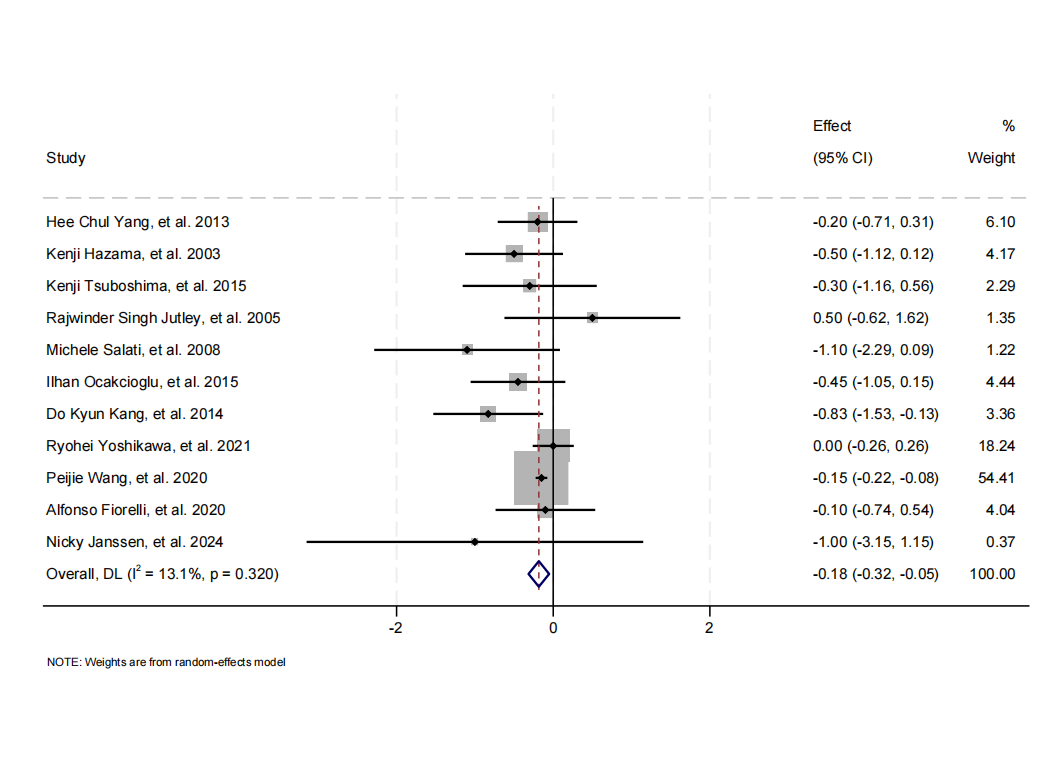
**

**Supplementary Figure S4b Forest plot of hospital stay comparing single-port versus multi-port VATS after excluding the wound-protector study (Song et al.)**

**Supplementary Figure S5 Sensitivity analysis of hospitalization time of single-hole and three-hole VATS pulmonary bullae resection**


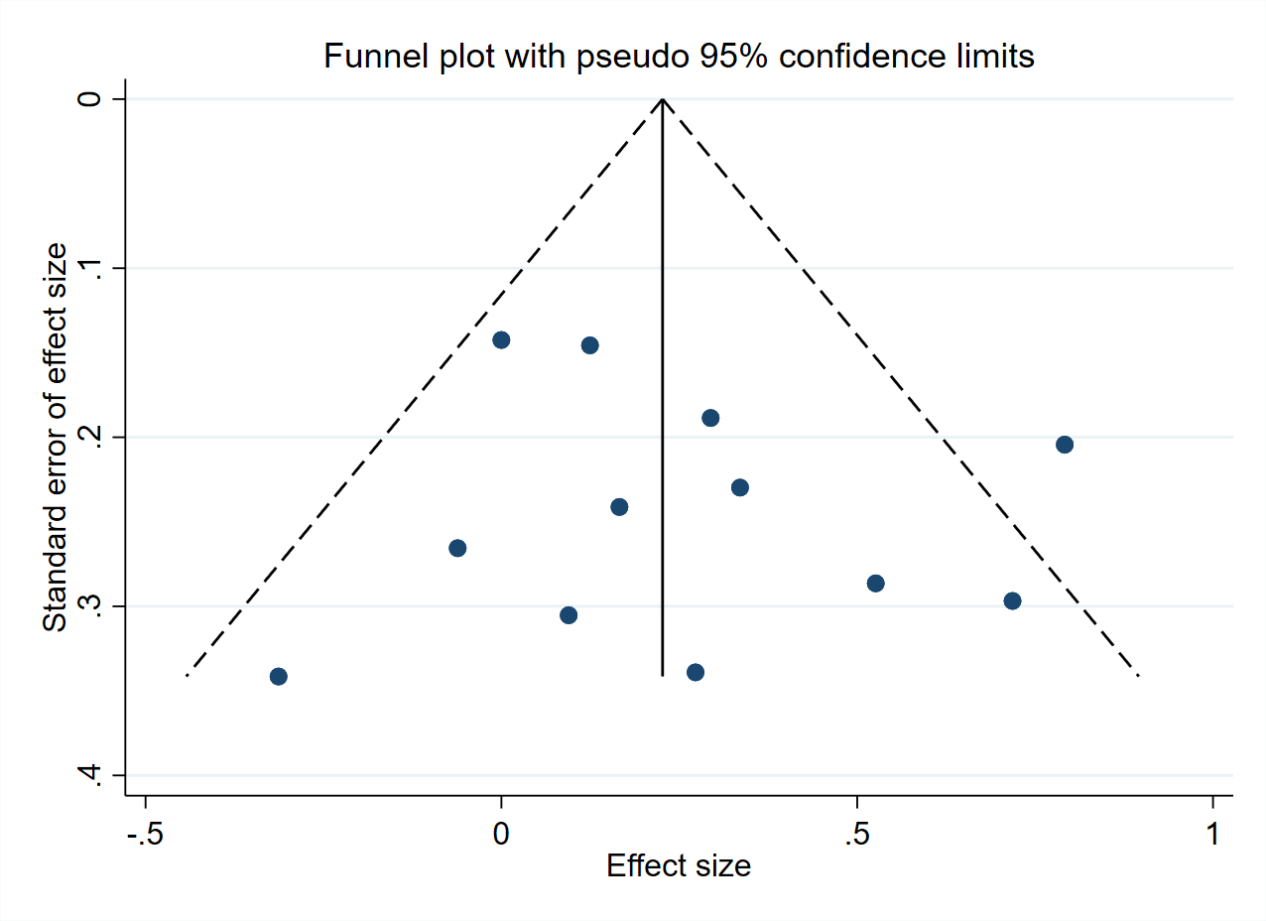


**Supplementary Figure S6 Funnel Plot for Hospital Stay Duration in Single-Port versus Triple-Port VATS for Bullous Lung Resection**


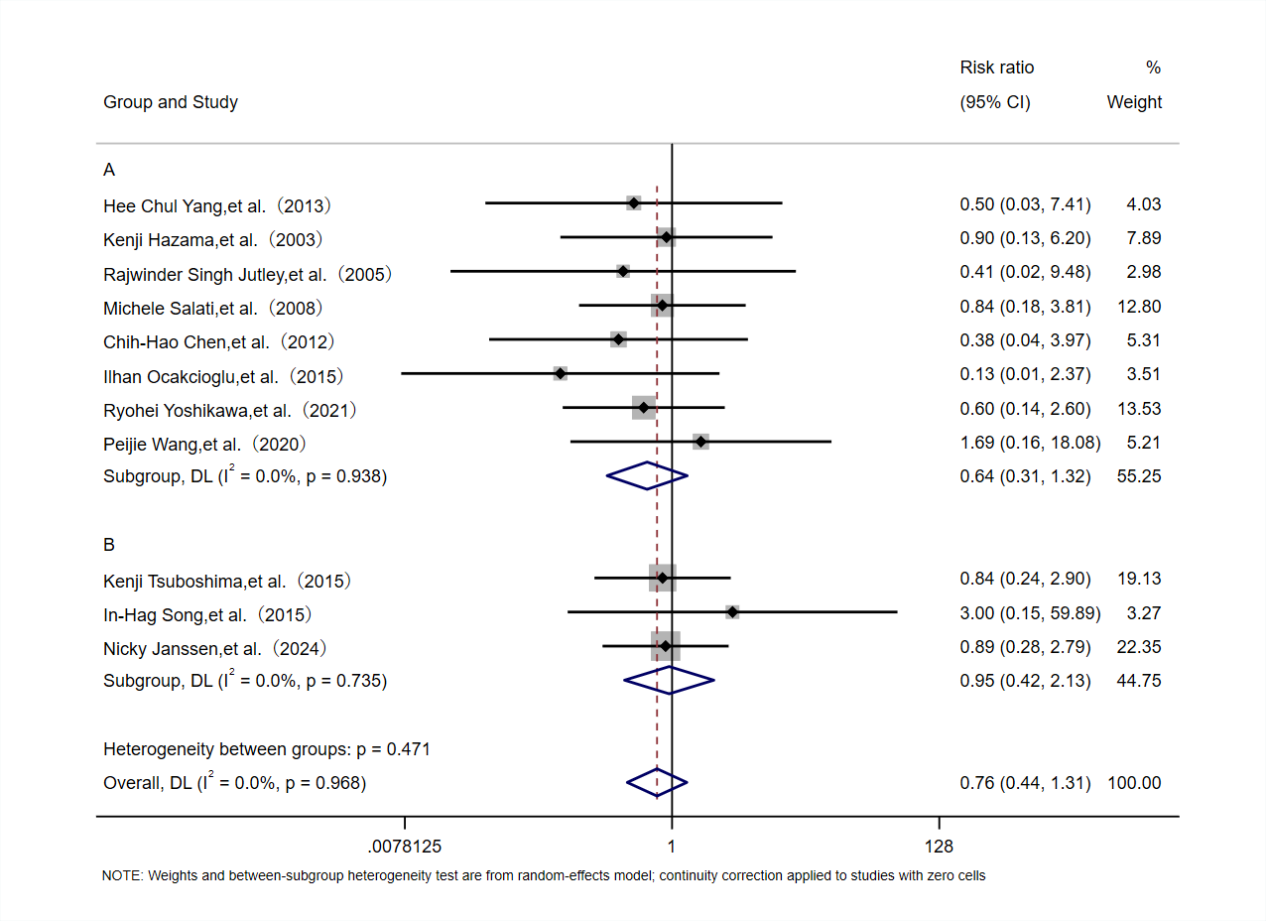


**Supplementary Figure S7 Comparison of postoperative recurrence between single-port and three-port VATS pulmonary bullae resection**

Group A : ordinary single-port VATS group ; group B : Modified uniportal VATS group

**Supplementary Figure S8 Sensitivity analysis of recurrence after single-hole and three-hole VATS pulmonary bullae resection**


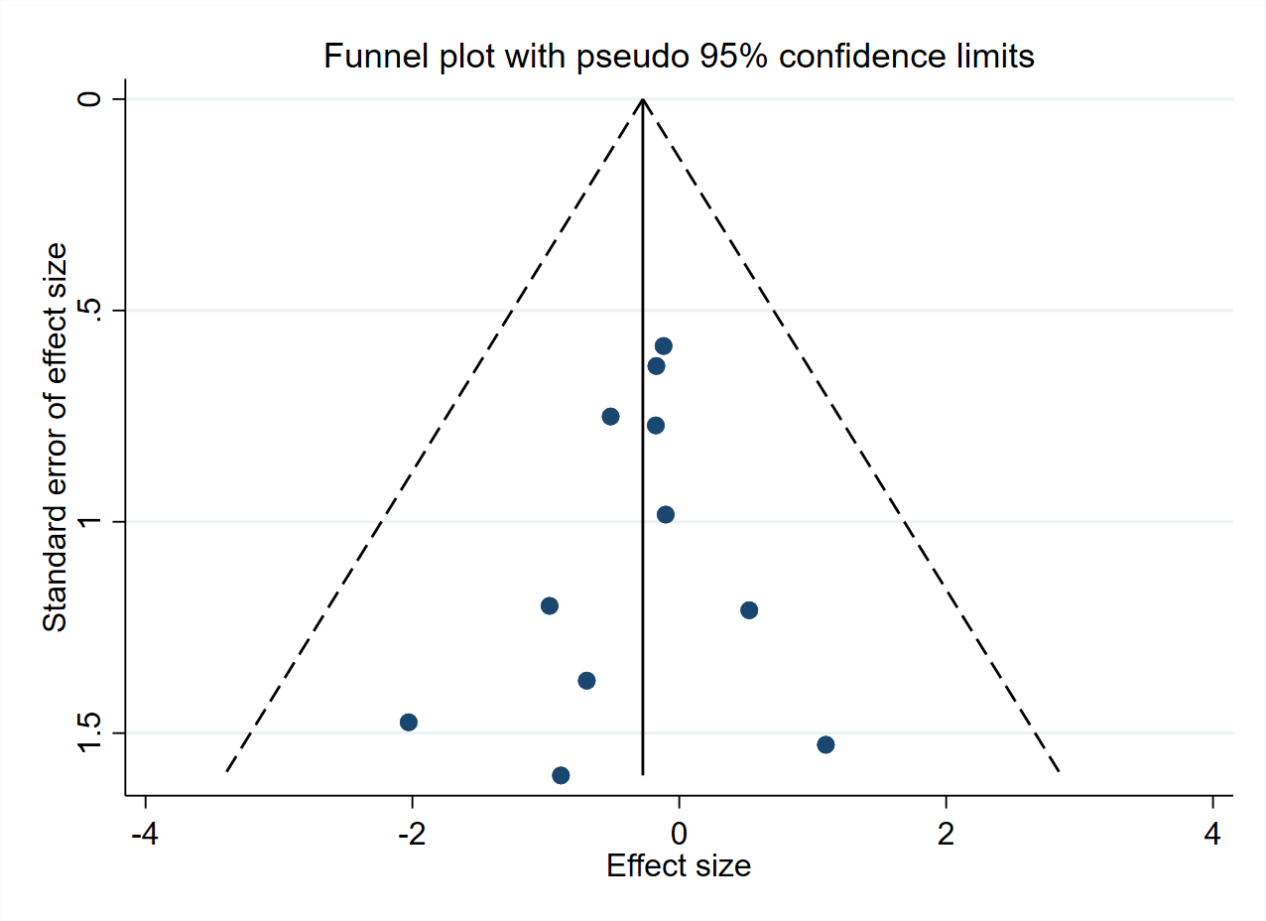


**Supplementary Figure S9 Funnel plot of recurrence after single-hole and three-hole VATS pulmonary bullae resection**


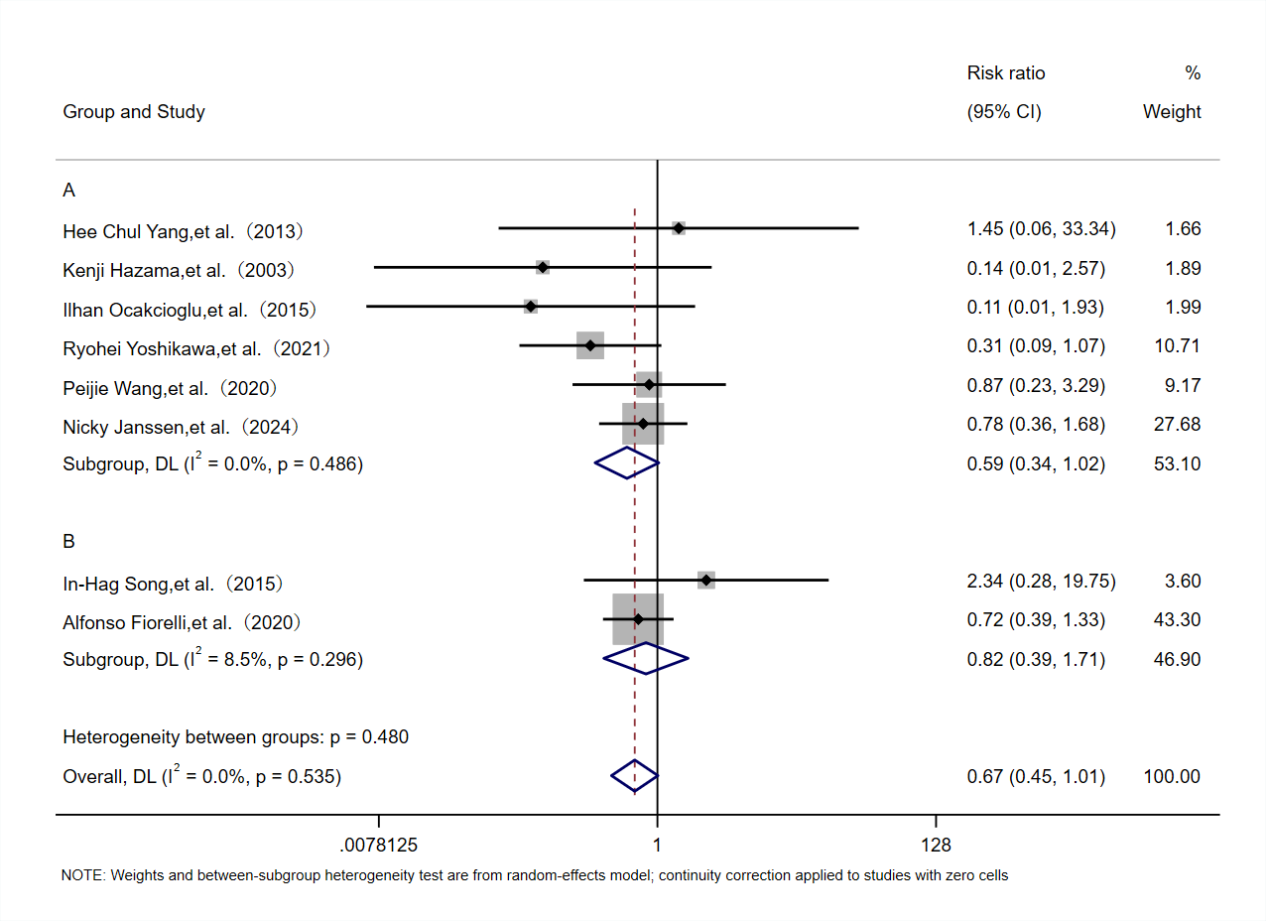


**Supplementary Figure S10 Comparison of postoperative complications between single-port and three-port VATS pulmonary bullae resection**

Group A : ordinary single-port VATS group ; group B : Modified uniportal VATS group

**Supplementary Figure S11 Sensitivity analysis of postoperative complications of single-hole and three-hole VATS pulmonary bullae resection**


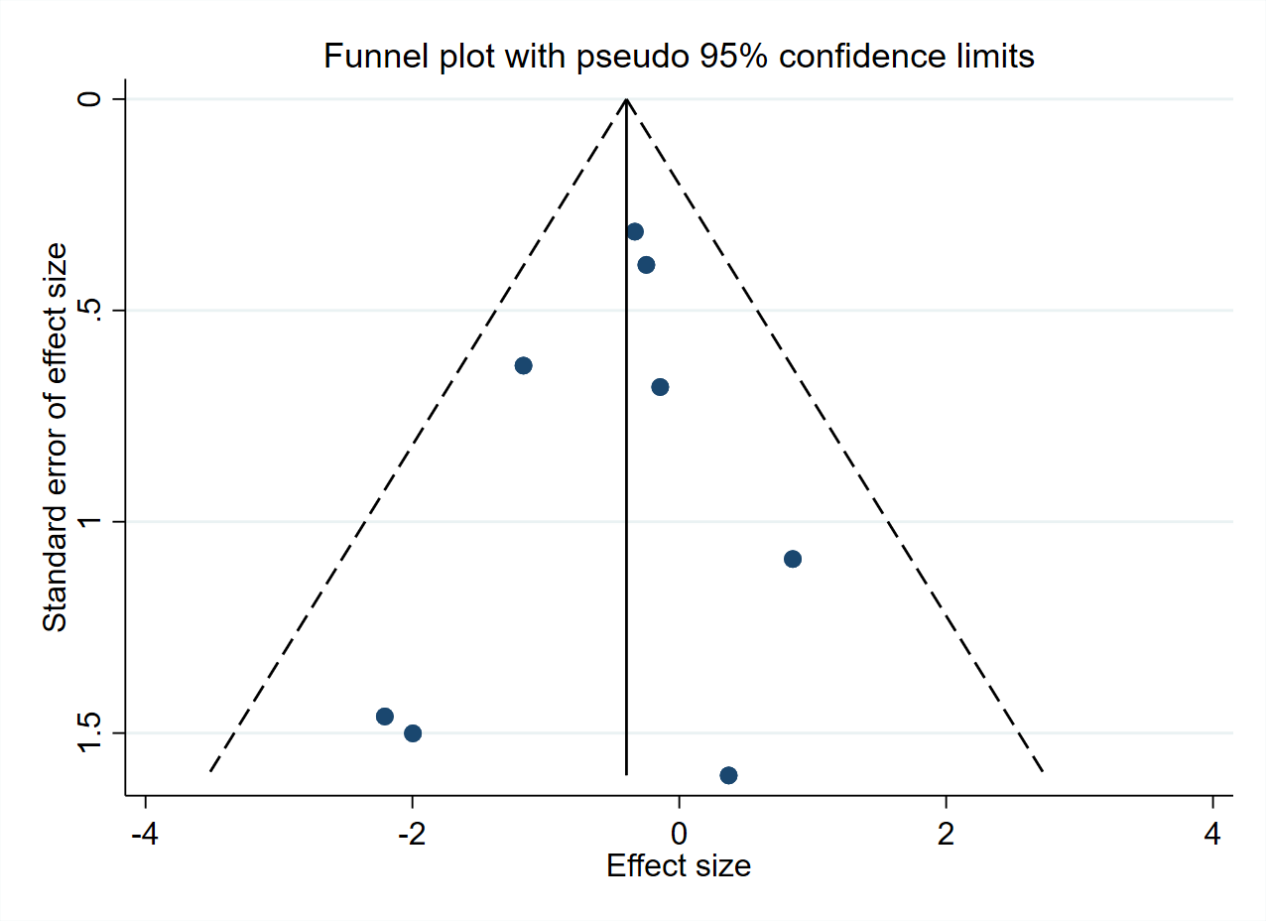


**Supplementary Figure S12 Publication bias risk funnel plot of postoperative complications of single-hole and three-hole VATS pulmonary bullae resection**


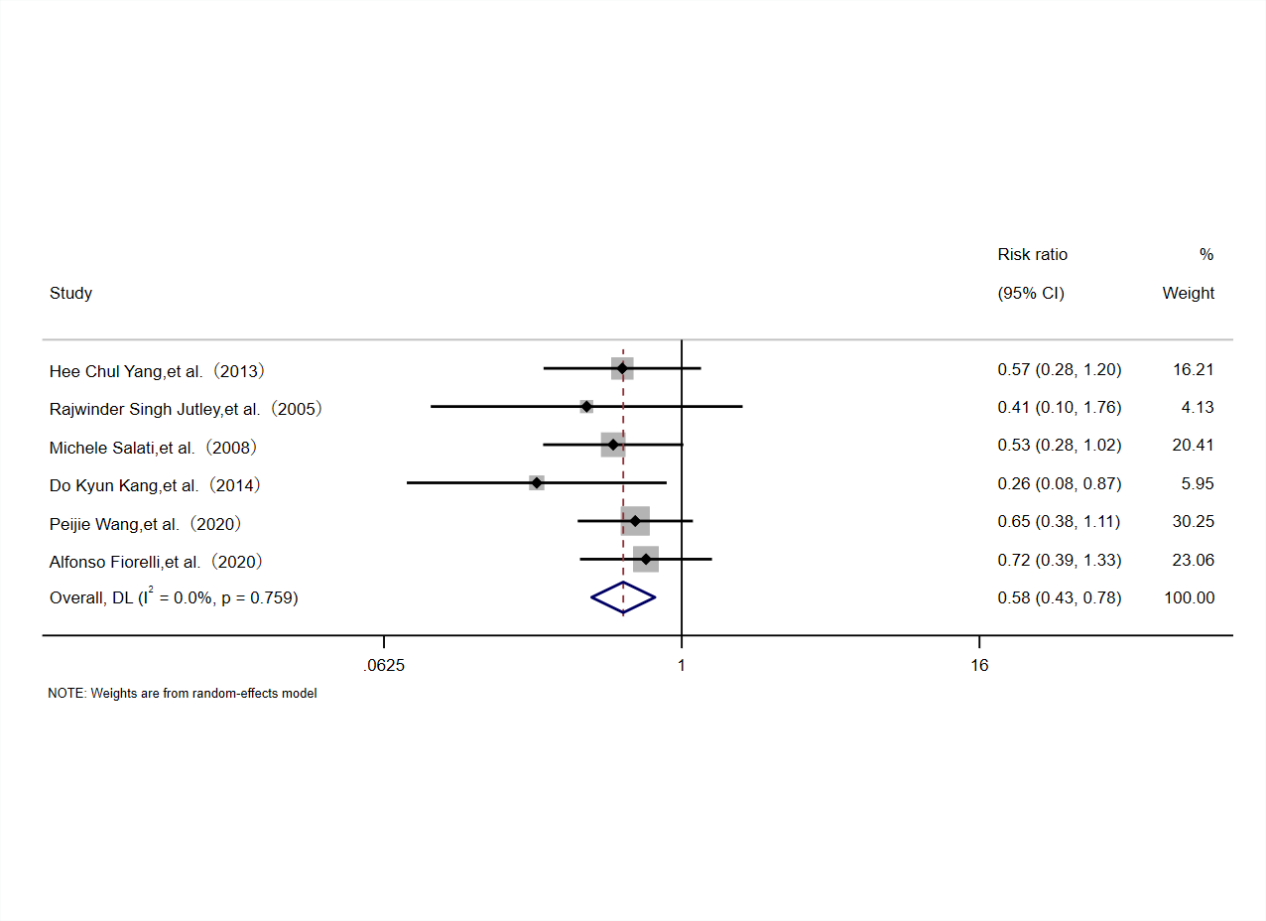


**Supplementary Figure S13 Comparison of chest wall paresthesia between single-hole and three-hole VATS pulmonary bullae resection**

**Supplementary Figure S14 Sensitivity Analysis for Chest Wall Paresthesia in Single-Port versus Triple-Port VATS for Bullous Lung Resection**


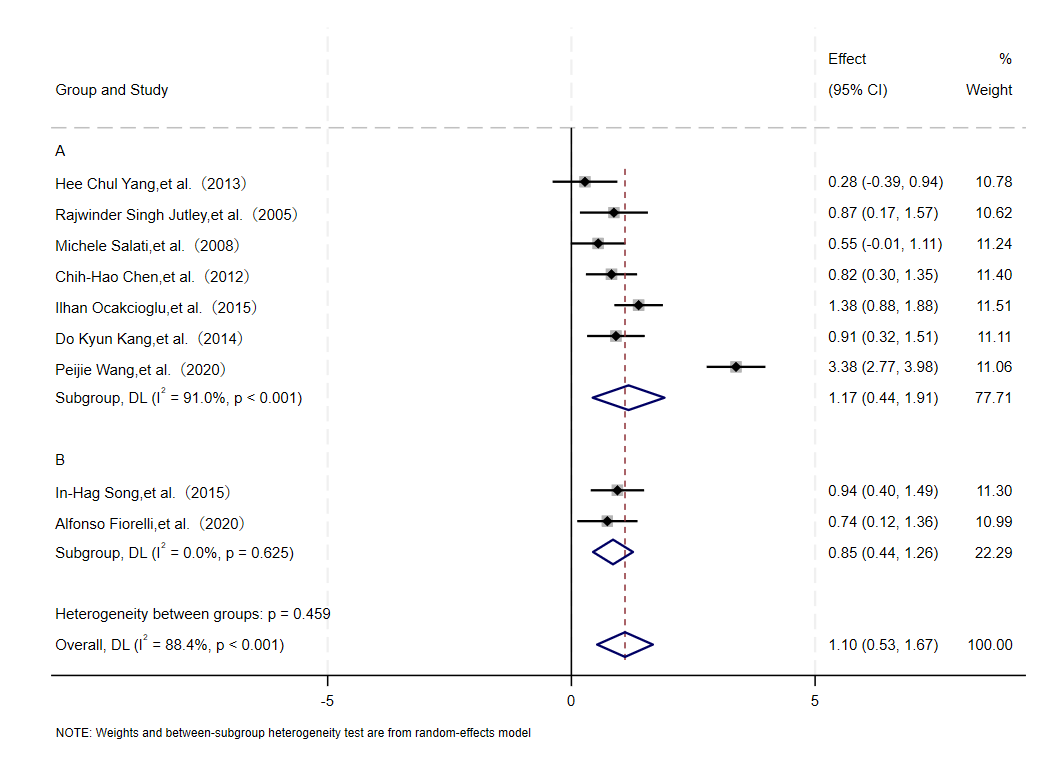


**Supplementary Figure S15 Comparison of postoperative pain between single-port and three-port VATS pulmonary bullectomy**

**Supplementary Figure S16 Analysis of pain sensitivity after single-hole and three-hole VATS pulmonary bullae resection**


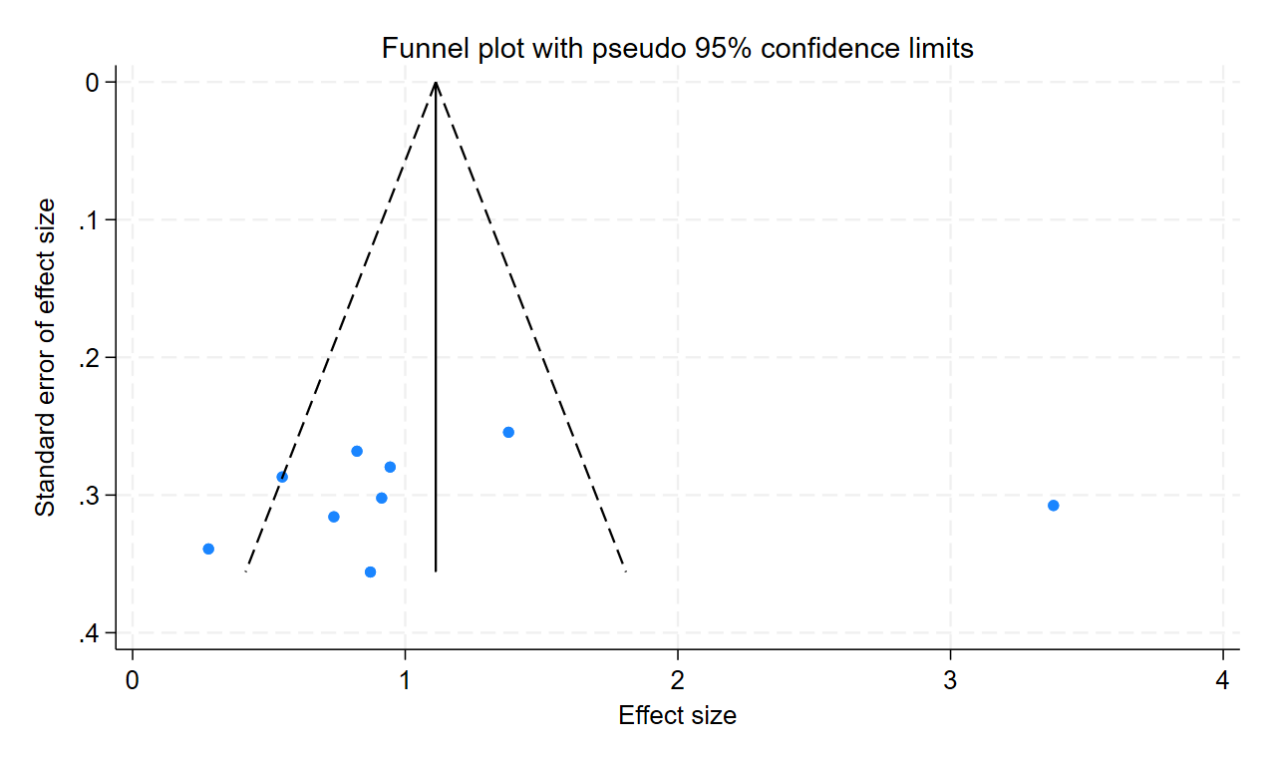


**Supplementary Figure S17 Funnel Plot for Publication Bias Risk of Postoperative Pain in Single-Port versus Triple-Port VATS for Bullous Lung Resection**


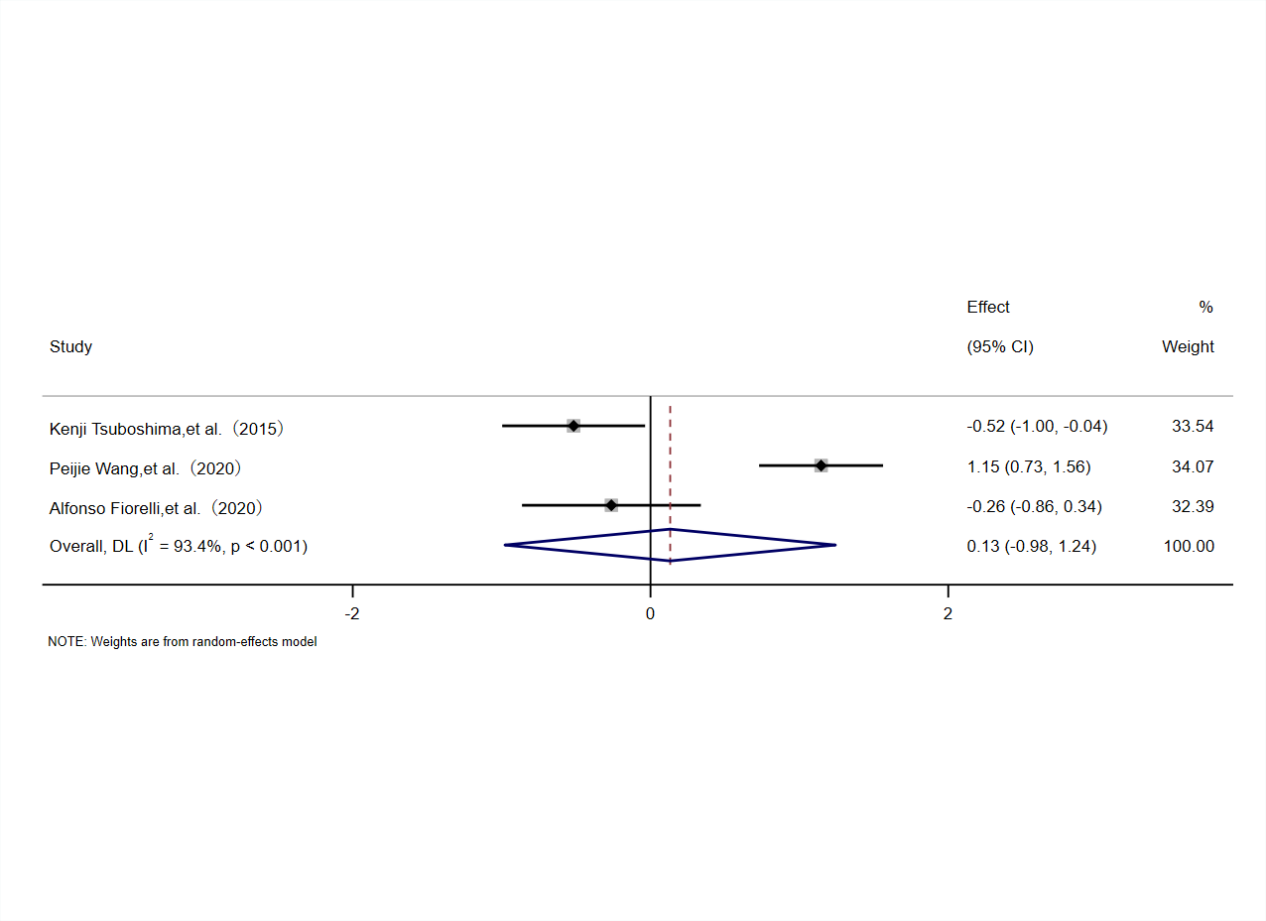


**Supplementary Figure S18 Comparison of the number of sutures in single-hole and three-hole VATS pulmonary bulla resection**


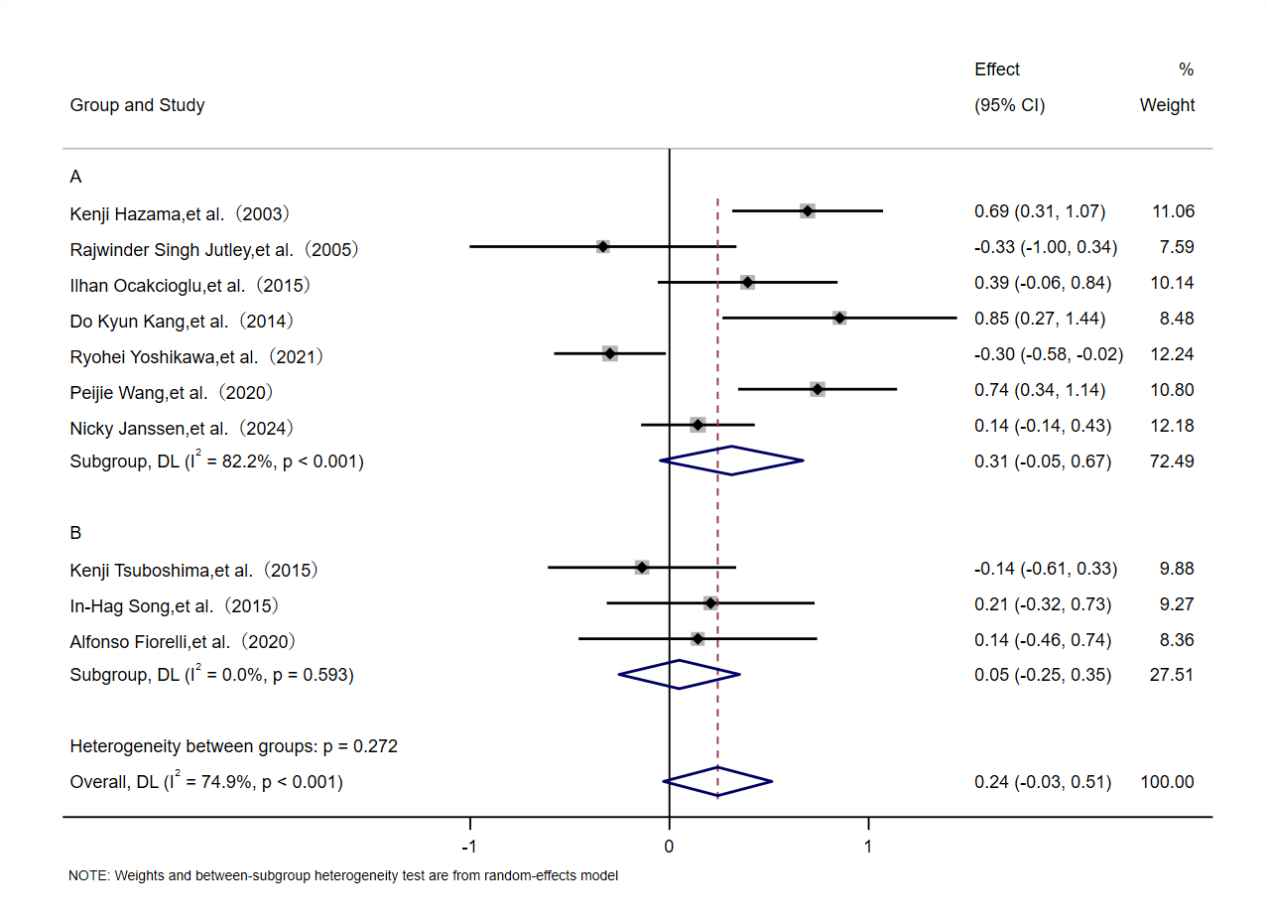


**Supplementary Figure S19 Comparison of Chest Tube Indwelling Time between Single-Port and Triple-Port VATS for Bullous Lung Resection**


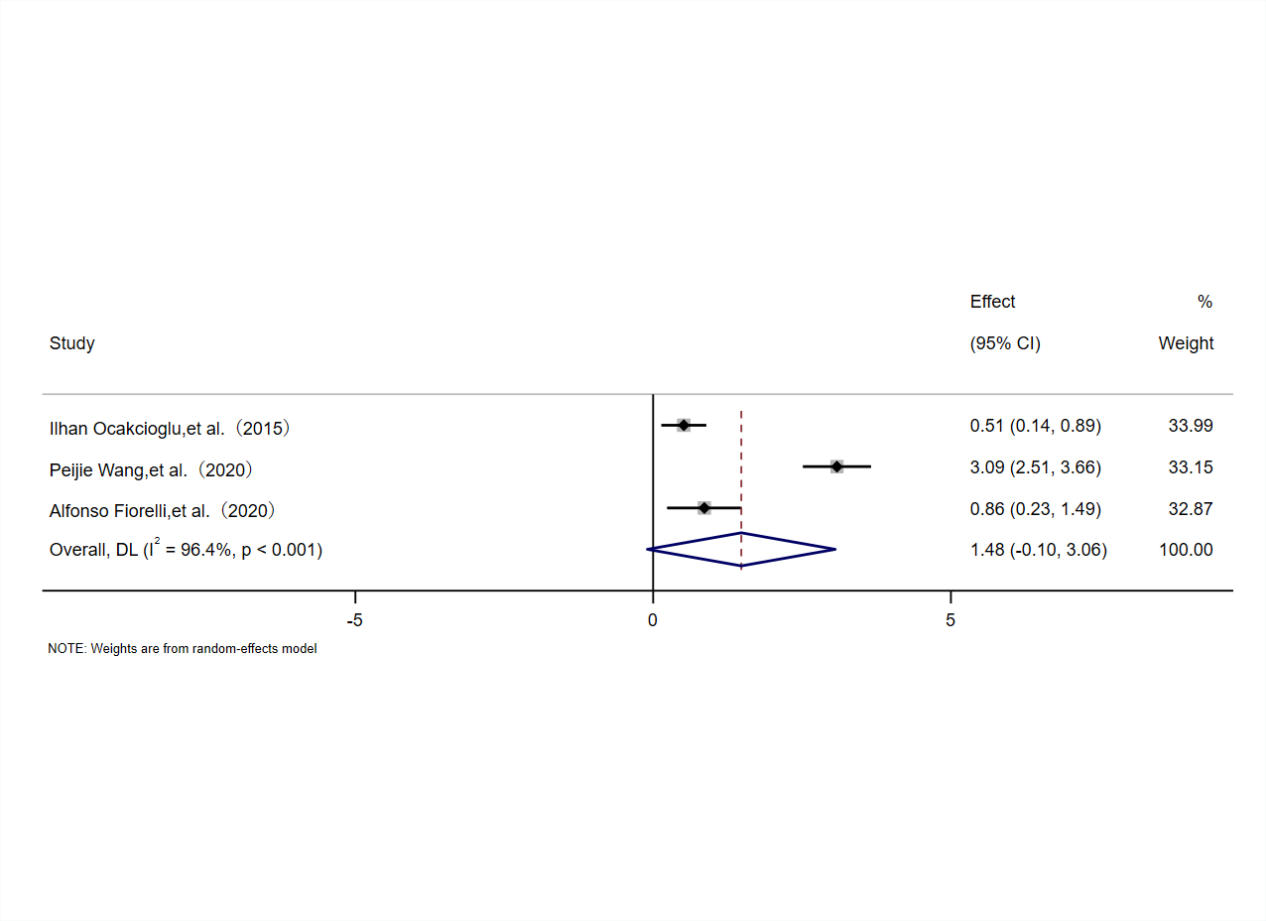


**Supplementary Figure S20 Comparison of patient satisfaction after single-hole and three-hole VATS pulmonary bullae resection**
